# Supplementary material for: The cancer cell proteome and transcriptome predicts sensitivity to targeted and cytotoxic drugs
Source: Life Sci Alliance. 2019 Jun 28;2(4):e201900445. doi: 10.26508/lsa.201900445 (PMC6600015; doi:10.26508/lsa.201900445)
Supplement: Supplementary file 1 [file Supplementary_Figures_Tables_Data_Sets_and_Source_Codes.zip › LSA-2019-00445_Description_of_the_Supplementary_Source_Data_Data_Set_Description_of_Data.pdf]

Supplementary materials: Signaling and expression states of cancer cells predict sensitivity to targeted and cytotoxic drugs

Tables

T1 `gt_mel_rppa_coefs.csv`

PLS (unnormalized) coefficients from (phospho)proteins to drug AUC in melanoma.

T2 `gt_mel_rnaseq_coefs.csv`

PLS (unnormalized) coefficients from gene expression to drug AUC in melanoma.

Source Data

D1 `Endometrial_Drug_Data_drugsel.csv`

In-house pharmacological data for endometrial cell lines.

D2 `Endometrial_RPPA_Data.csv`

In-house proteomic (RPPA) data for endometrial cell lines.

D3 `GNE208 RPPA Data.csv`

In-house proteomic (RPPA) data for melanoma cell lines.

D4 `Melanoma_Endometrial_RNASeq.csv`

In-house transcriptomic data for melanoma and endometrial cell lines.

D5 `Melanoma Sensitivity Data.csv`

In-house pharmacological data for melanoma cell lines.

D6 `GDC0068.csv`

In-house pharmacological data for GDC0068 in melanoma cell lines.

D7 `melanoma_vcf_matrix_all.csv`

In-house processed genomic data for melanoma cell lines.

D8 `melanoma_vcf_matrixAnyPtMut_all.csv`

In-house processed genomic data for melanoma cell lines.

D9 `melanoma_vcf_matrixGeneMut_all.csv`

In-house processed genomic data for melanoma cell lines.

D10 `prot2ensg_ccle.csv`

Convert CCLE protein names to Ensemble identifiers.

D11 `prot2sym_gt_skin.csv`

Convert in-house protein names to gene symbols.

D12 `speed1_default.txt`

SPEED1 signature genes for different pathways (**?**).

D13 `drug_targets_ccle_curated.csv`

Drug target annotation for a subset of drugs within CCLE.

D14 `drug_targets_ctrp_curated.csv`

Drug target annotation for a subset of drugs within CTRP.
