# Supplementary material for: The cancer cell proteome and transcriptome predicts sensitivity to targeted and cytotoxic drugs
Source: Life Sci Alliance. 2019 Jun 28;2(4):e201900445. doi: 10.26508/lsa.201900445 (PMC6600015; doi:10.26508/lsa.201900445)
Supplement: Supplementary file 1 [file Supplementary_Figures_Tables_Data_Sets_and_Source_Codes.zip › LSA-2019-00445_Supplemental_Material_Figure_S1-S23.pdf]

Supplemental Information: Signaling and expression states of cancer cells predict sensitivity to targeted and cytotoxic drugs

1 Cross-validation yields negative correlation for unpredictive models

For several drugs, a negative Spearman rank correlation was observed between predicted and measured drug sensitivity. The explanation for this is that when there is no relationship between the input and output variables to be found the model effectively predicts drug sensitivity as an average of the training data. Mathematically, when the predicted sensitivity $\hat{S}_i$ for cell line i is given by the average of the other $N-1$ cell line sensitivities we have [1]

$$\hat{S}_i = \frac{1}{N-1} \sum_{j=1, j \neq i}^{N} S_j = \frac{N}{N-1} \bar{S} - \frac{1}{N-1} S_i$$

showing that the predicted sensitivity $\hat{S}_i$ is in fact perfectly anti-correlated with the measured sensitivity S_i. A strong negative Spearman rank correlation does not, however, necessarily imply a strong negative fraction of variance explained $R^2 = 1 - \frac{\langle (\hat{S}_i - S_i)^2 \rangle}{\langle (S_i - \bar{S})^2 \rangle}$.

[1] http://not2hastie.tumblr.com/

2 Supplemental Figures

Unless stated otherwise figures reflect results in our in-house melanoma cell line panel. Unless stated otherwise PLS was used to train models on transcriptomic/proteomic data and regression trees to train models on genomic data as described in Methods.

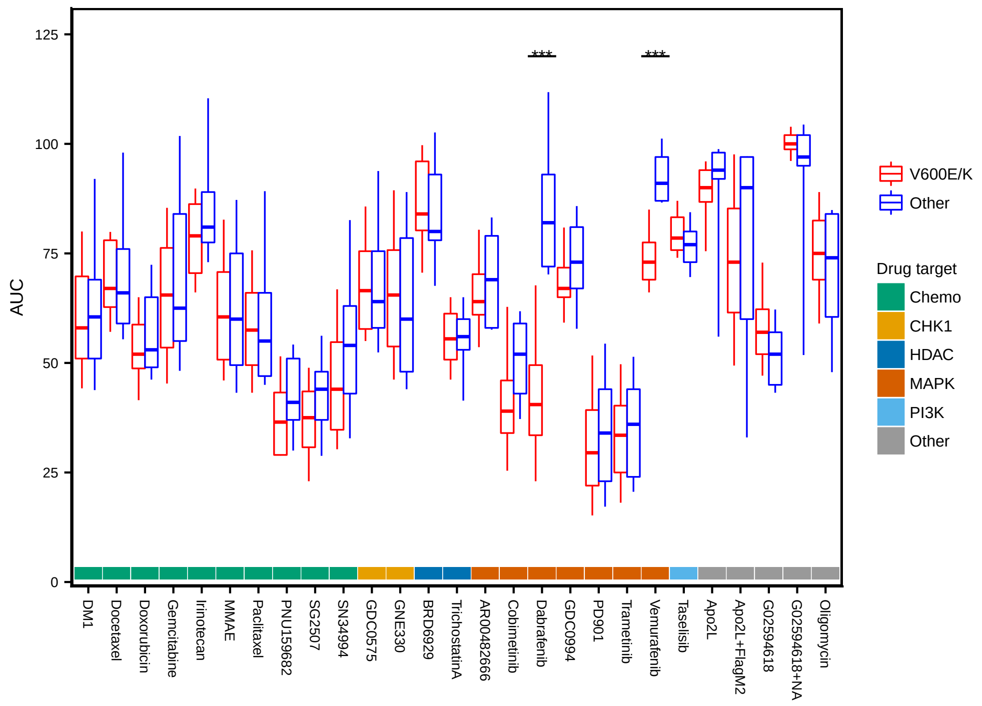

Figure S1: Drug AUC shown separately for cell lines with or without BRAF$^{\mathrm{V600E/K}}$ mutation. Only the BRAF inhibitors Vemurafenib and Dabrafenib show a differential response between the two groups (Benjamini-Hochberg adjusted $p = 4.2 \cdot 10^{-7}$ and $p = 1.9 \cdot 10^{-5}$).

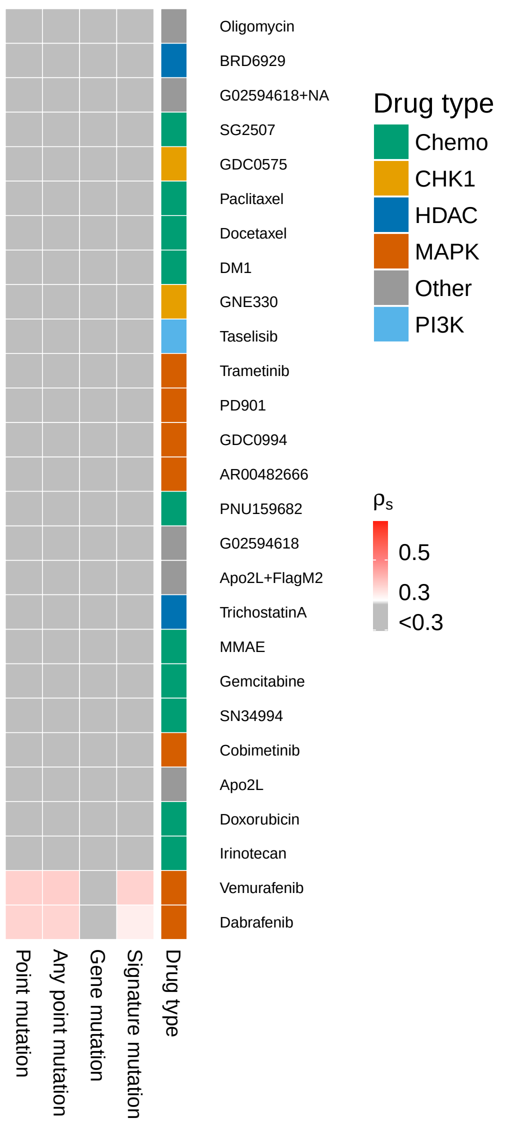

Figure S2: Predicting drug AUC from genomic data in melanoma using different definitions of "mutation". *Point mutation* considers both location and new base pair identity. *Any point mutation* considers only location of mutation but not new base pair identity. *Gene mutation* considers any point mutation at any location in a gene. *Signature mutation* considers only $BRAF^{V600E/K}$, BRAF (other), $NRAS^{G12/Q61}$, and KRAS mutations.

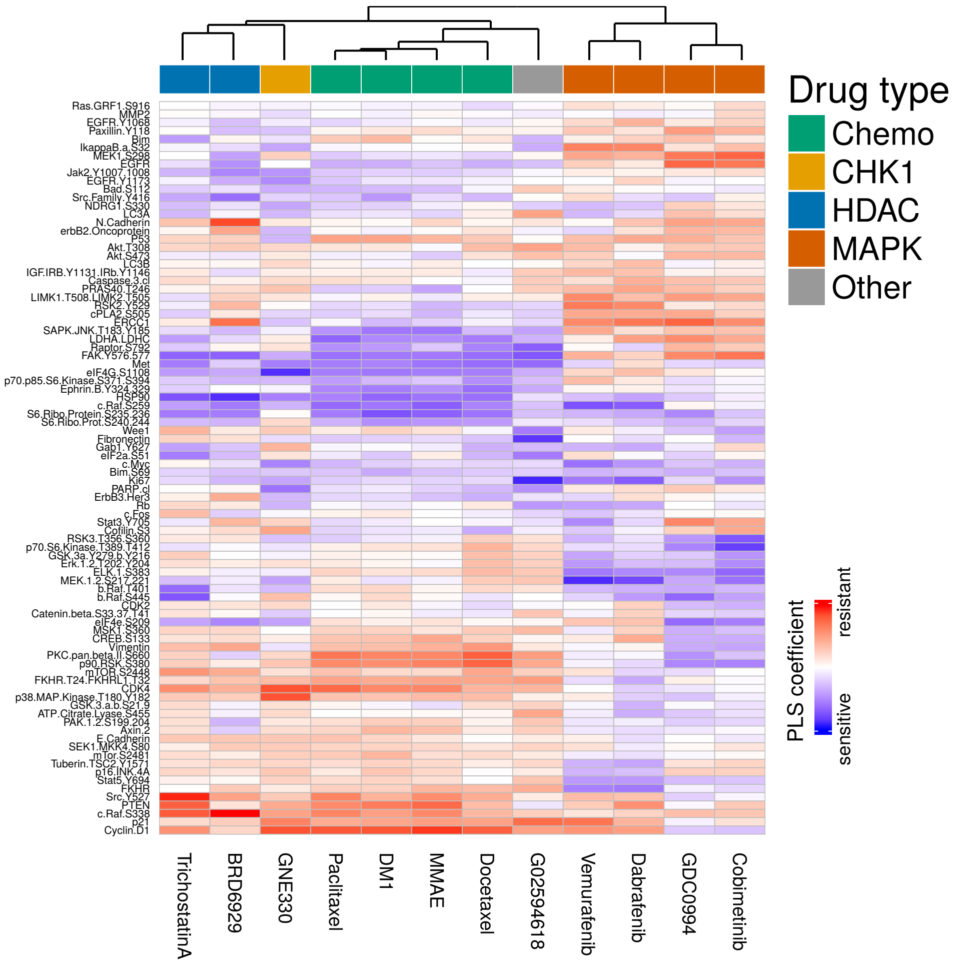

Figure S3: PLS models from proteomic data to drug AUC. The PLS coefficients suggest associations between (phospho)proteins and drug sensitivity or resistance. Unsupervised hierarchical clustering of the PLS models predominantly clusters drugs sharing common targets.

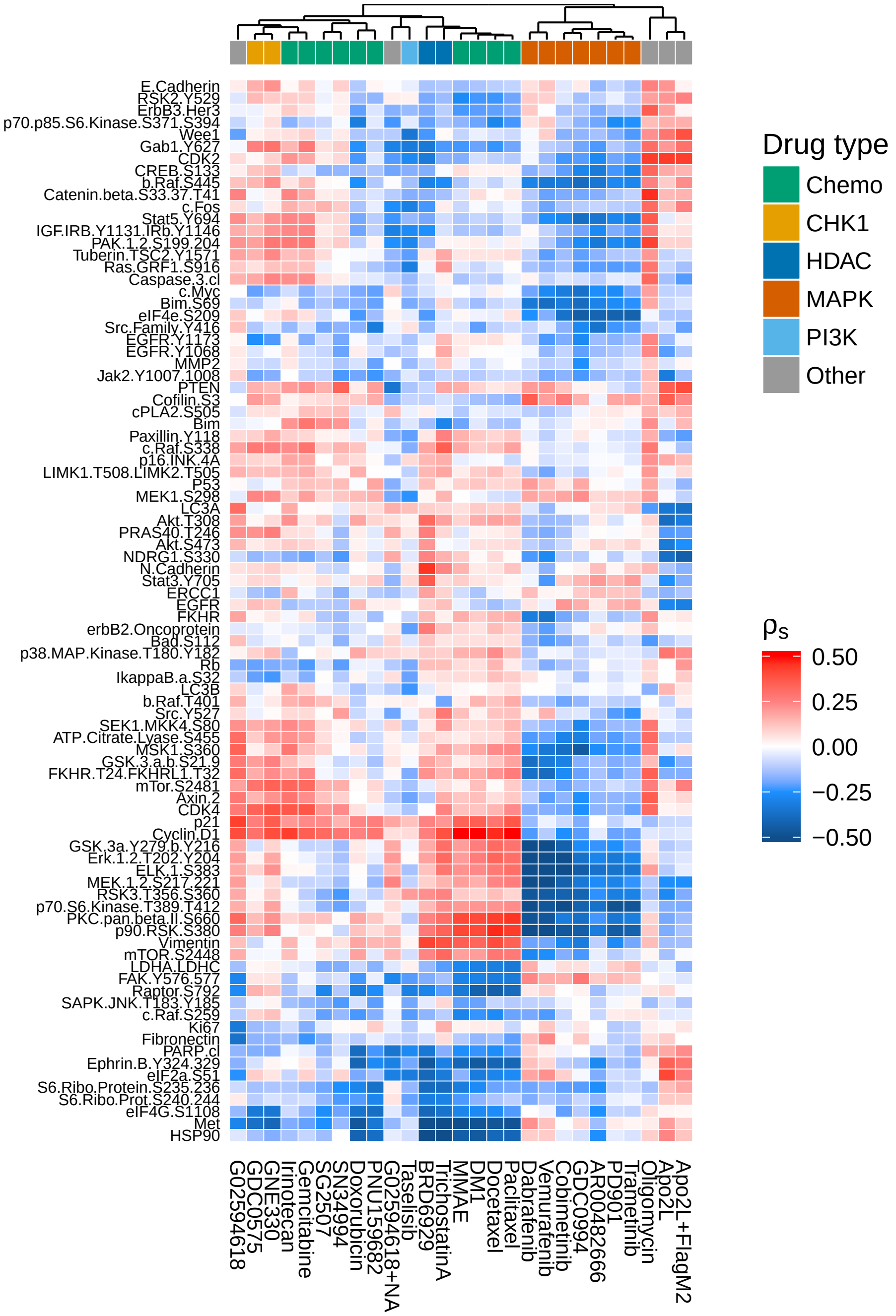

Figure S4: Spearman rank correlation between drug AUC and individual (phospho)proteins.

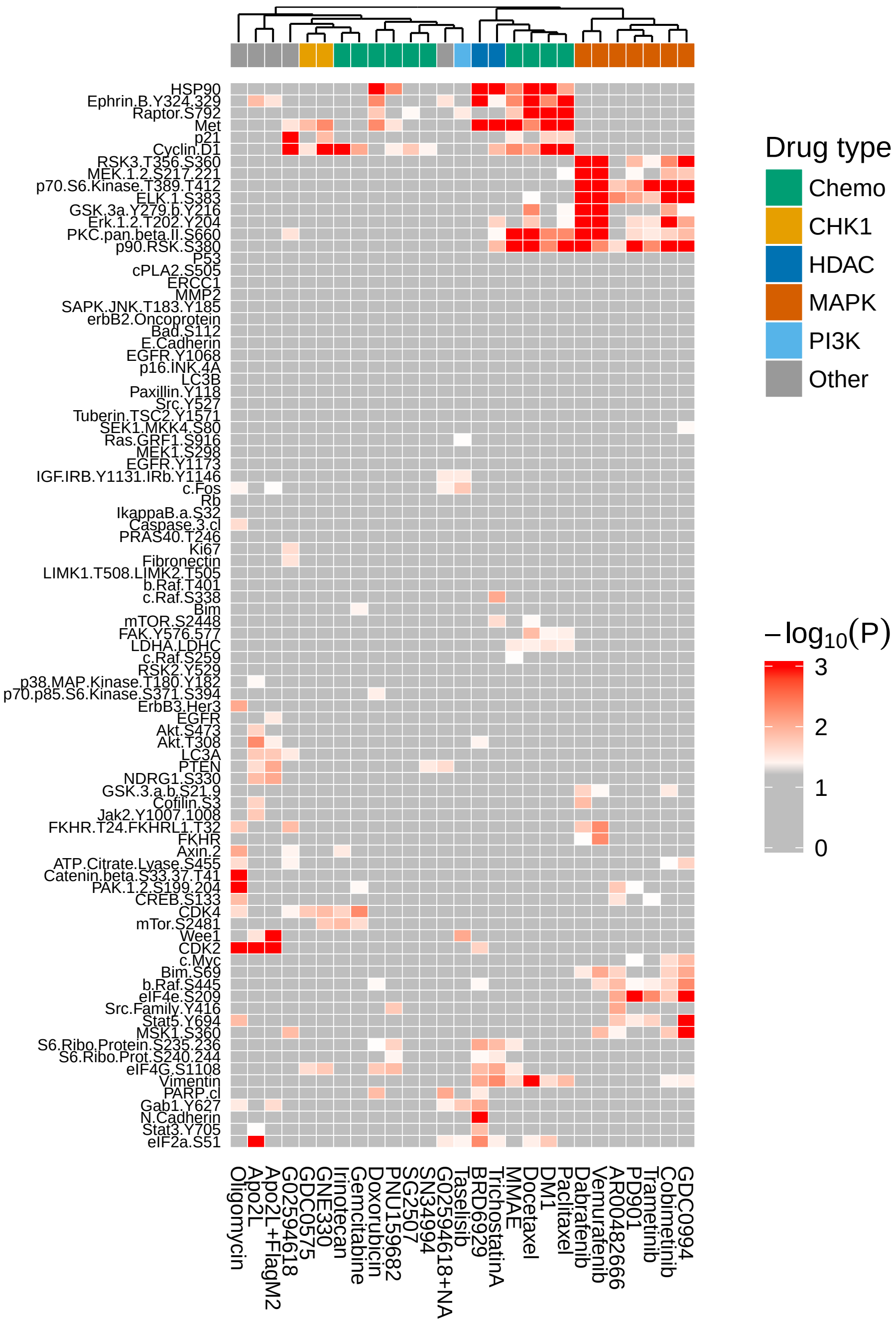

Figure S5: p-values for Spearman rank correlation between drug AUC and individual (phospho)proteins with respect to random shuffling of the AUCs for each drug ($N = 1024$).

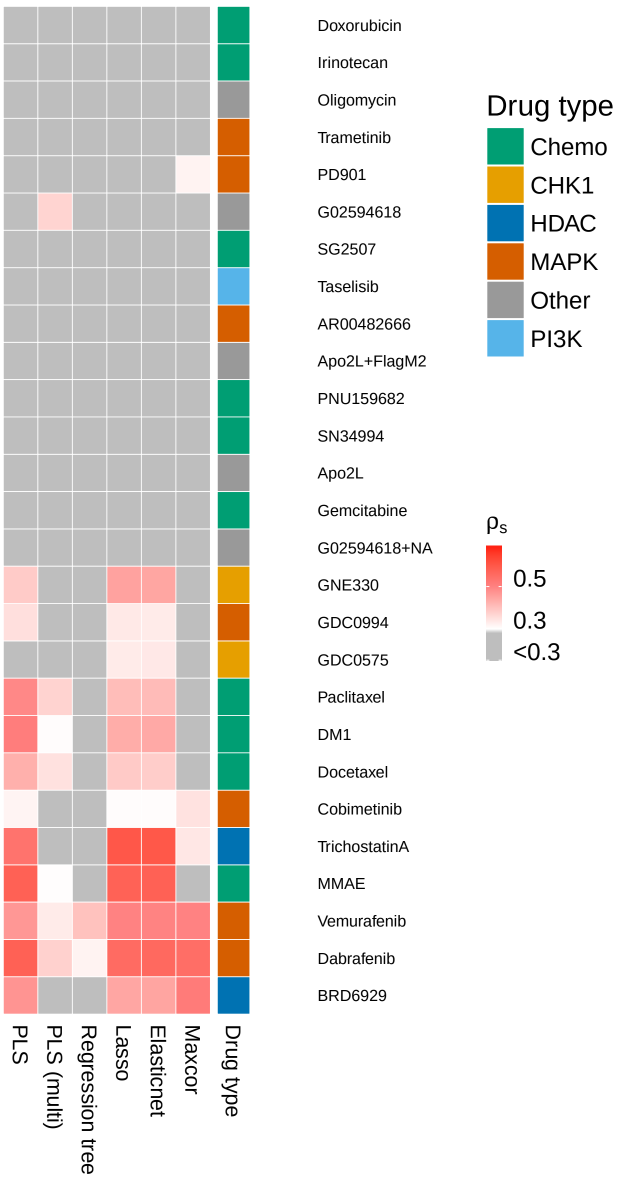

Figure S6: Predicting drug AUC from proteomic data using different algorithms.

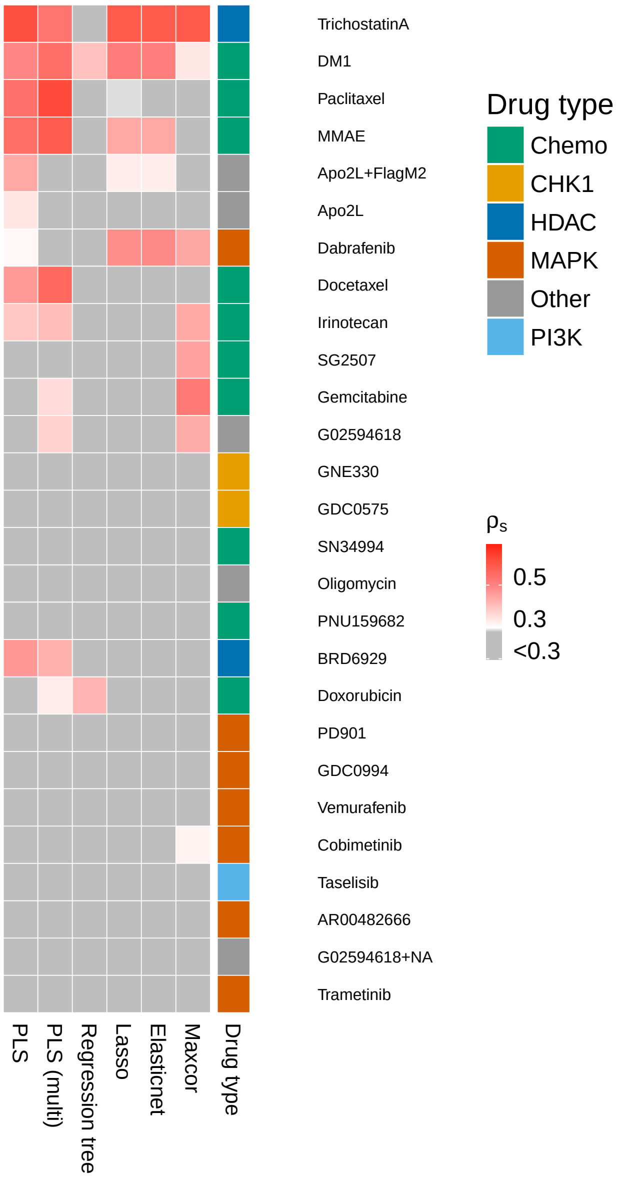

Figure S7: Predicting drug AUC in melanoma from transcriptomic data using different algorithms.

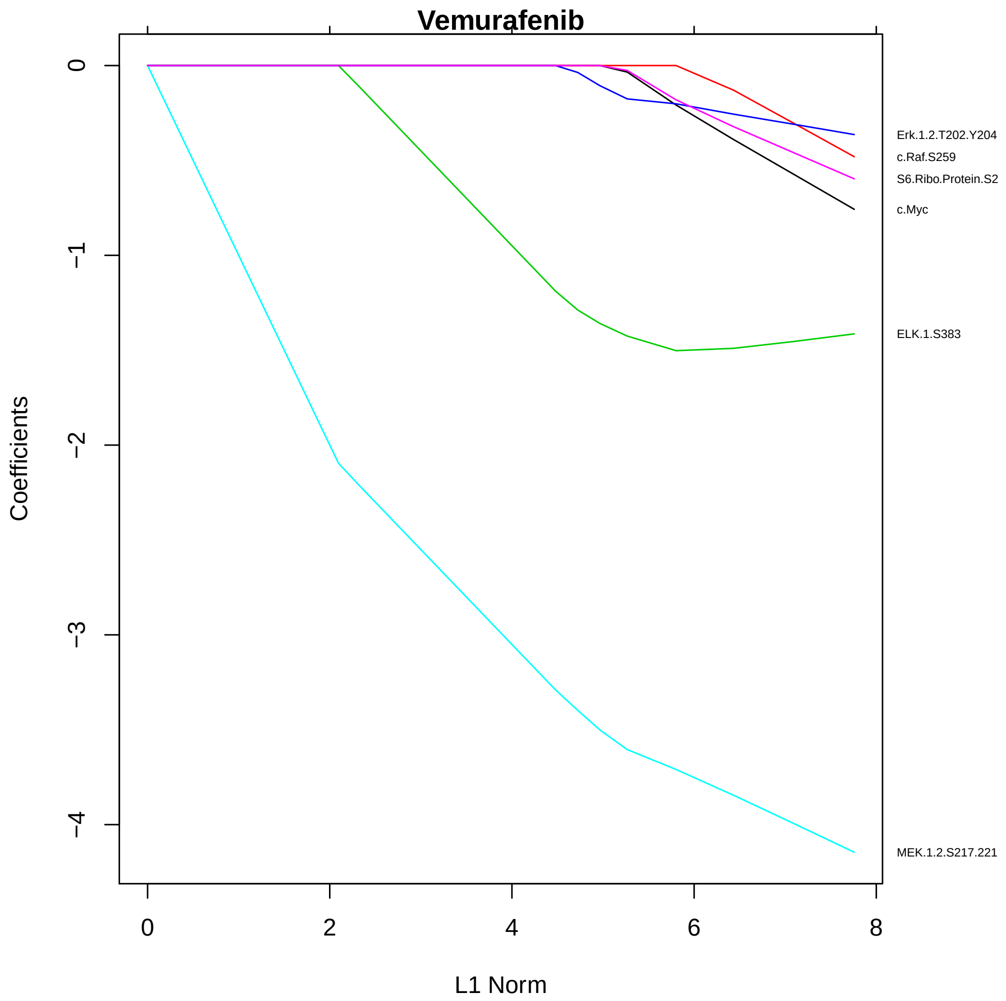

Figure S8: LASSO coefficient profile plot for Vemurafenib AUC prediction using proteomic data. A small L1 norm corresponds to a large penalty λ for non-zero coefficients in the regression model.

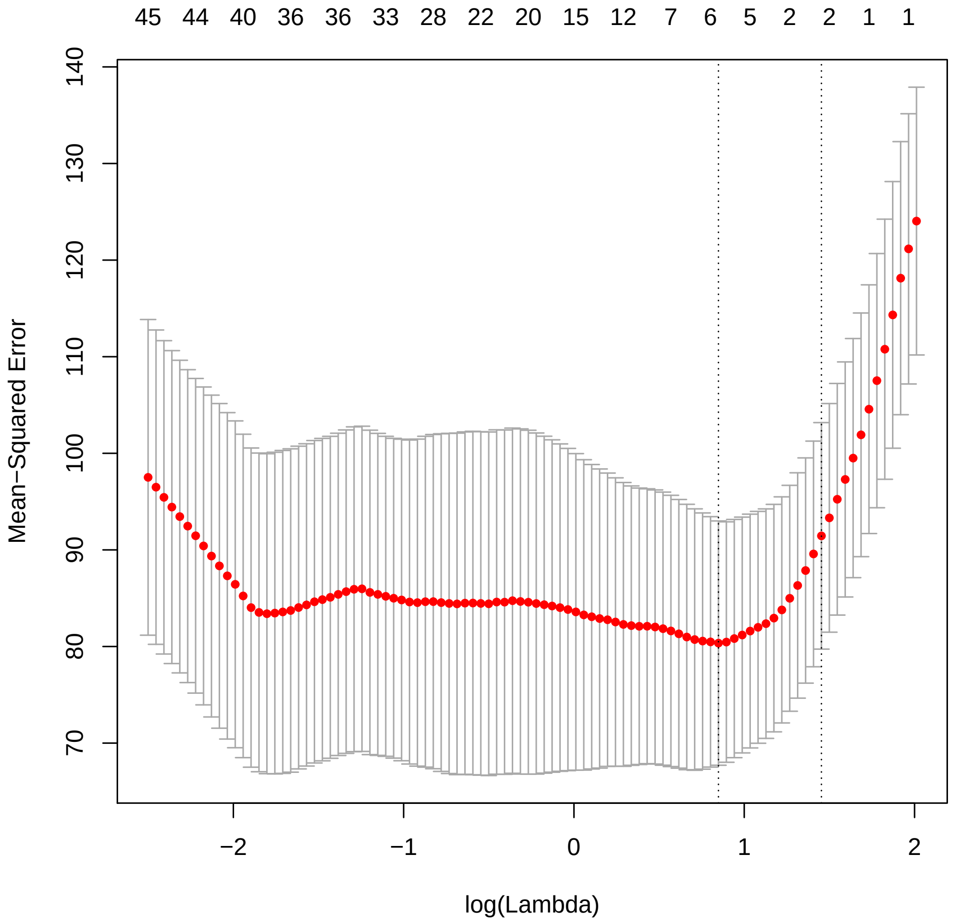

Figure S9: LASSO cross-validation error plot for Vemurafenib AUC prediction using proteomic data. The penalty λ for non-zero coefficients in the regression model is indicated on the x-axis. The number of non-zero coefficients for a given λ is shown above the plot. The left vertical line corresponds to the λ value which results in minimum error, and the right vertical line corresponds to the largest value of lambda such that the error is within one standard-error of the minimum error.

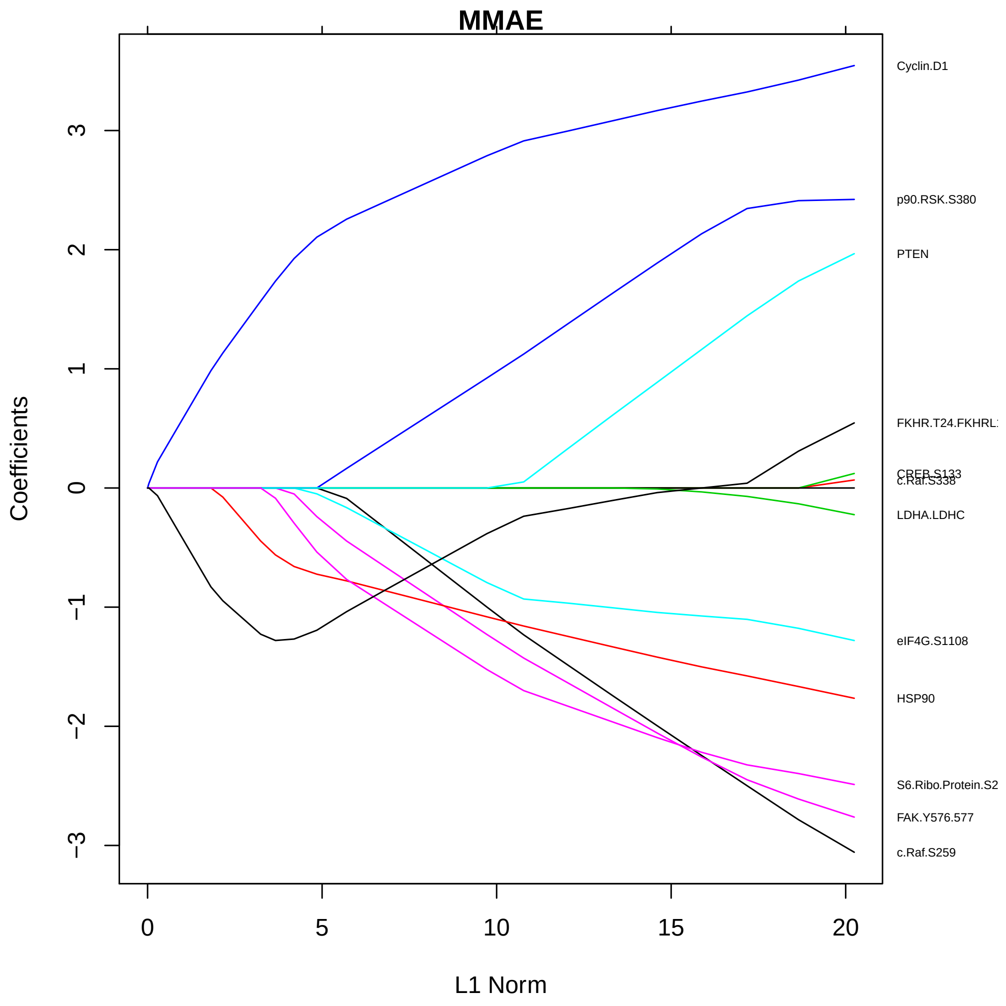

Figure S10: LASSO coefficient profile plot for MMAE AUC prediction using proteomic data. A small L1 norm corresponds to a large penalty λ for non-zero coefficients in the regression model.

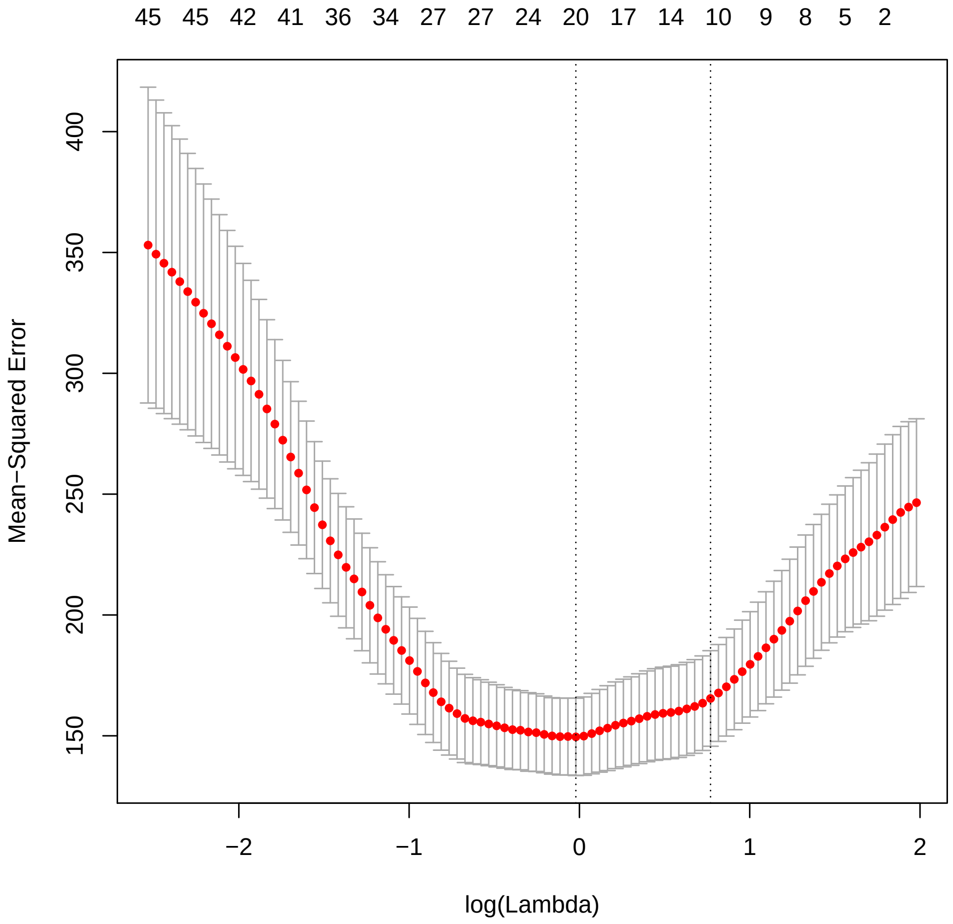

Figure S11: LASSO cross-validation error plot for MMAE AUC prediction using proteomic data. The penalty λ for non-zero coefficients in the regression model is indicated on the x-axis. The number of non-zero coefficients for a given λ is shown above the plot. The left vertical line corresponds to the λ value which results in minimum error, and the right vertical line corresponds to the largest value of lambda such that the error is within one standard-error of the minimum error.

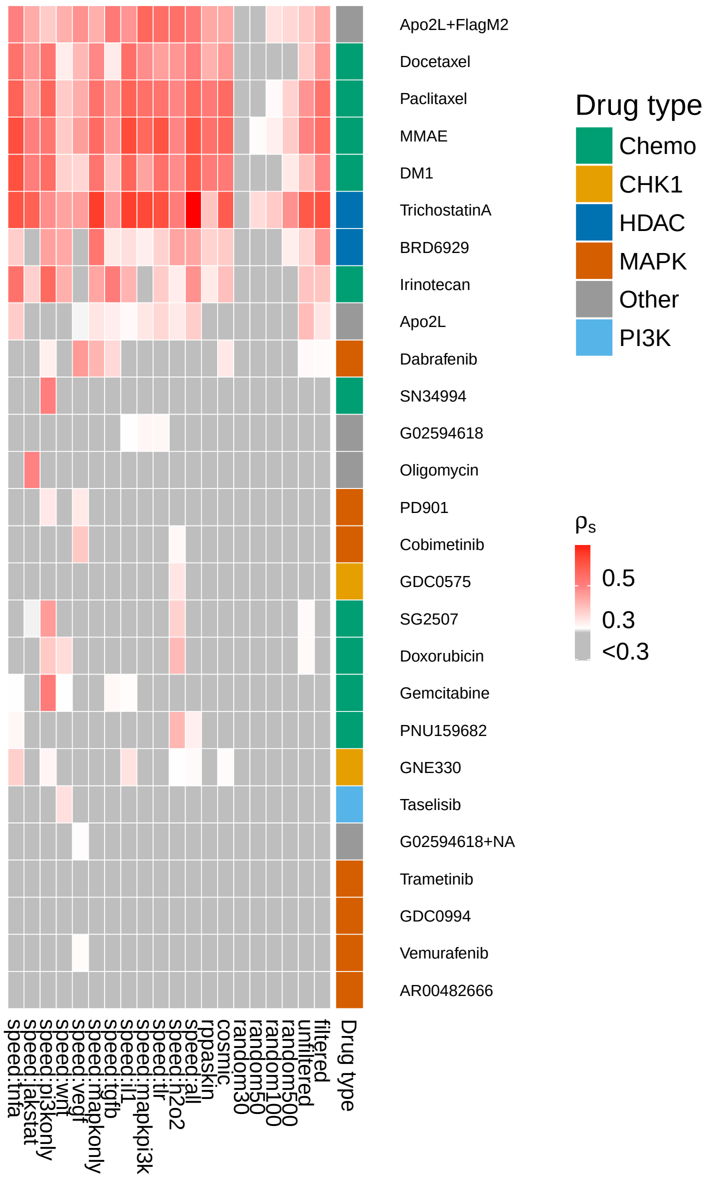

Figure S12: Predicting drug AUC from transcriptomic data using genes differentially expressed in key cellular pathways as reported by SPEED (Parikh *et al*, 2010), genes measured in the proteomic dataset, genes in the COSMIC Cancer Gene Census (Forbes *et al*, 2015), randomly selected genes, unfiltered genes, and mean+variance filtered genes.

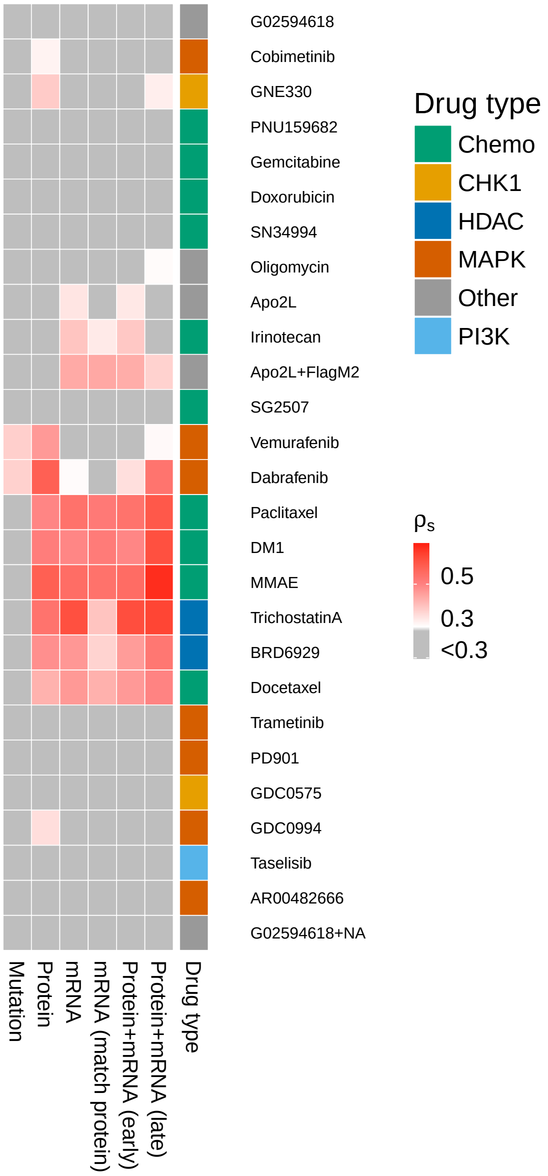

Figure S13: Drug AUC predictions based on genomic, transcriptomic, proteomic, or transcriptomic+proteomic data combined using either early or late integration.

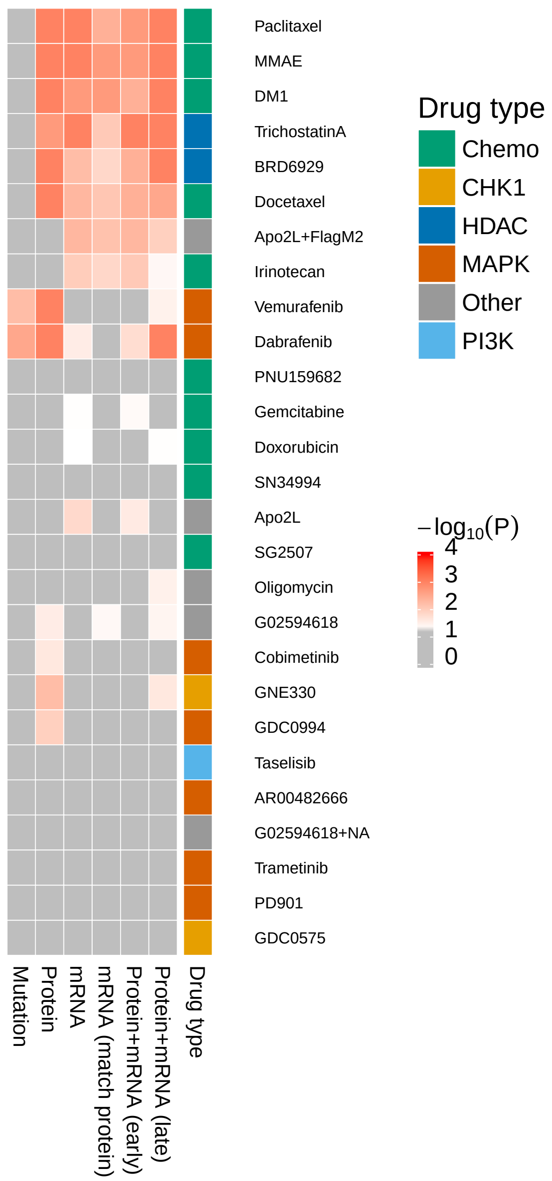

Figure S14: Drug AUC predictions based on genomic, transcriptomic, proteomic, or transcriptomic+proteomic data combined using either early or late integration.

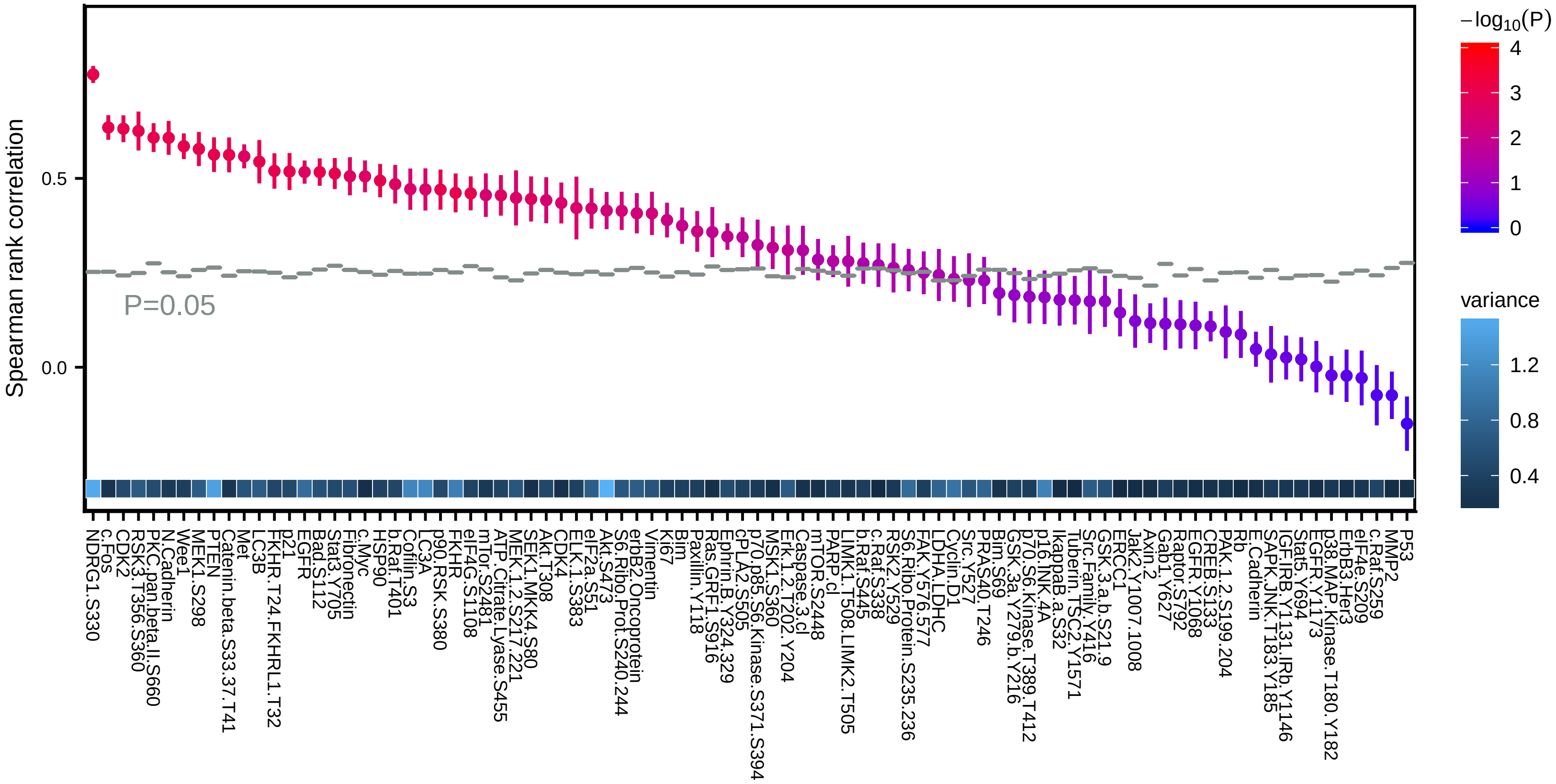

Figure S15: Predicting proteomic data from transcriptomic data. To reduce the computational time, transcriptomic data was reduced to genes reported in the COSMIC Cancer Gene Census (Forbes *et al*, 2015). According Supplemental Figures S12+S22, this gene subset predicts drug sensitivity equally well as compared to unfiltered transcriptomic data both in our melanoma panel and in CCLE data.

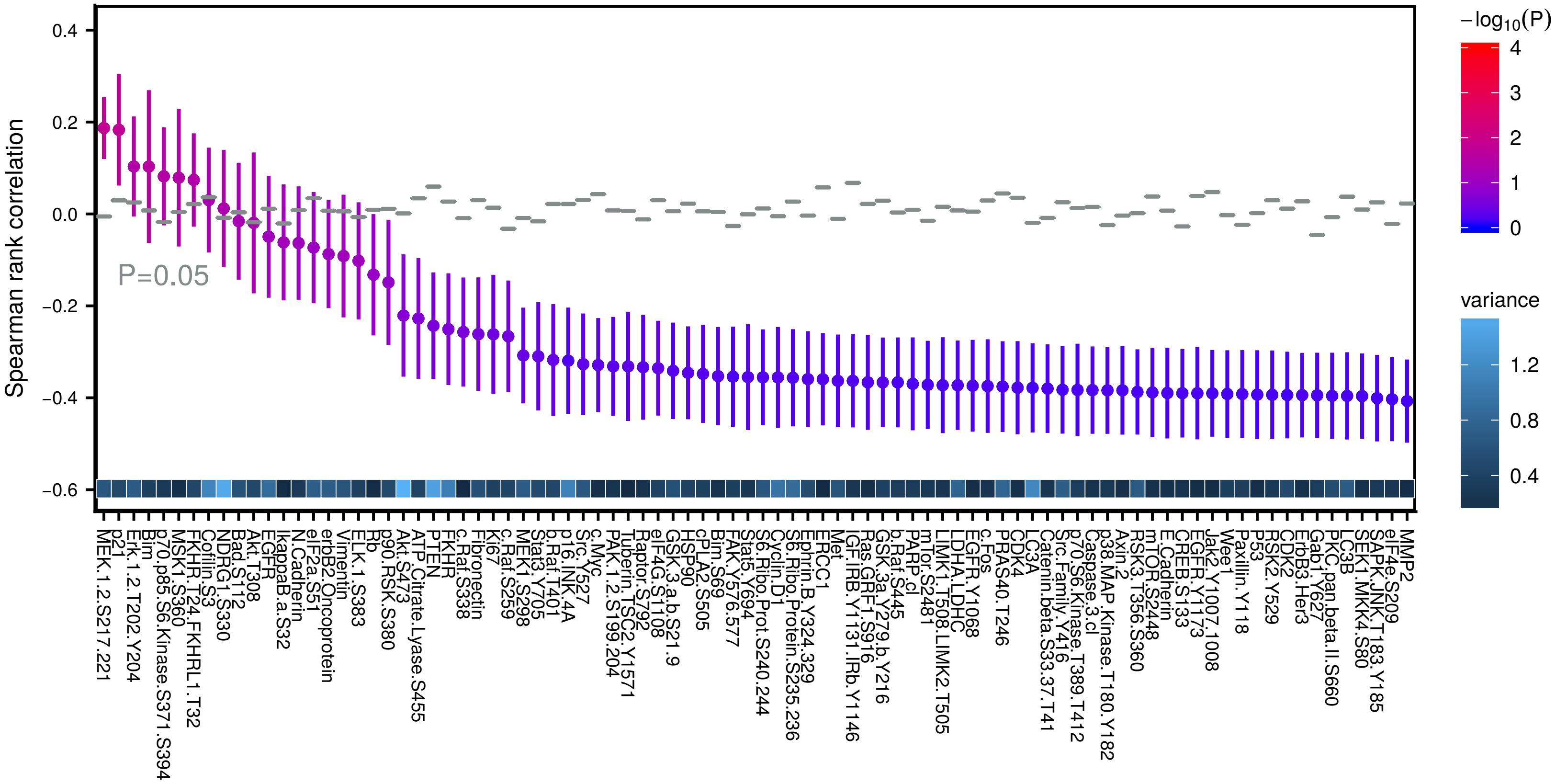

Figure S16: Predicting proteomic data from genomic data.

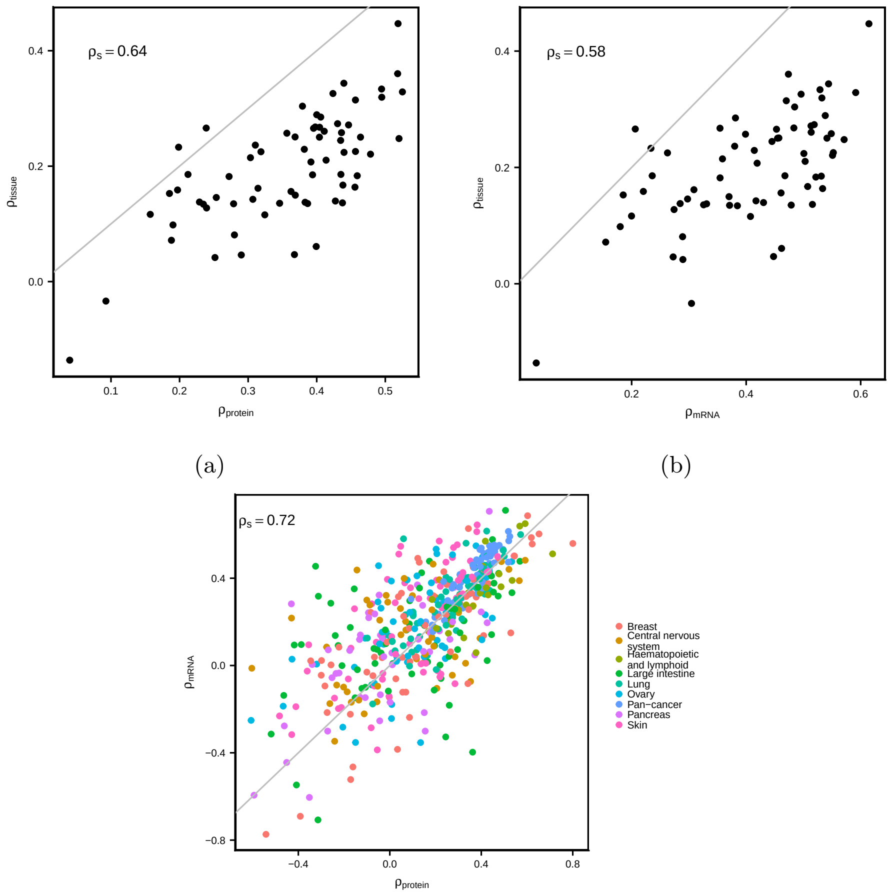

(a) (b)

(c)

Figure S17: Drug sensitivity predictions using CCLE data (Barretina *et al*, 2012) with two different datasets plotted against each other **a)** tissue type vs proteomic data **b)** tissue type vs transcriptomic data **c)** transcriptomic vs proteomic data

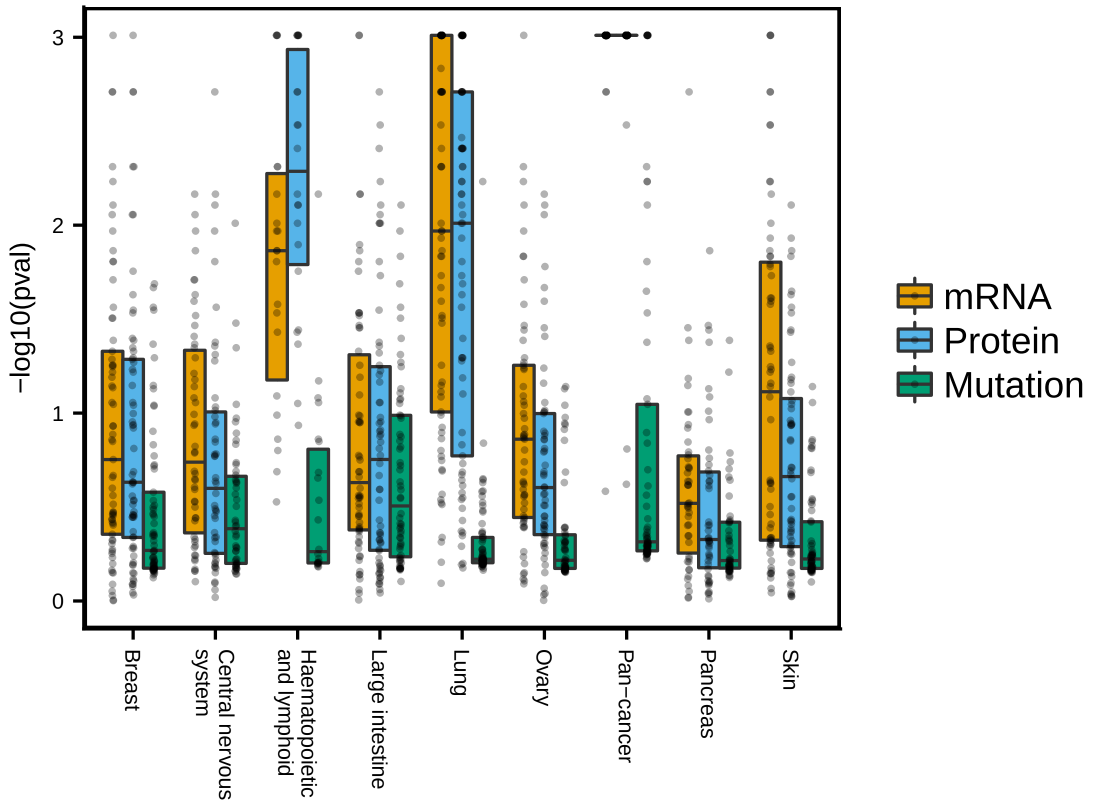

Figure S18: P-values for Spearman rank correlation between predicted and measured drug sensitivity after cross-validation using CCLE data (Barretina *et al*, 2012).

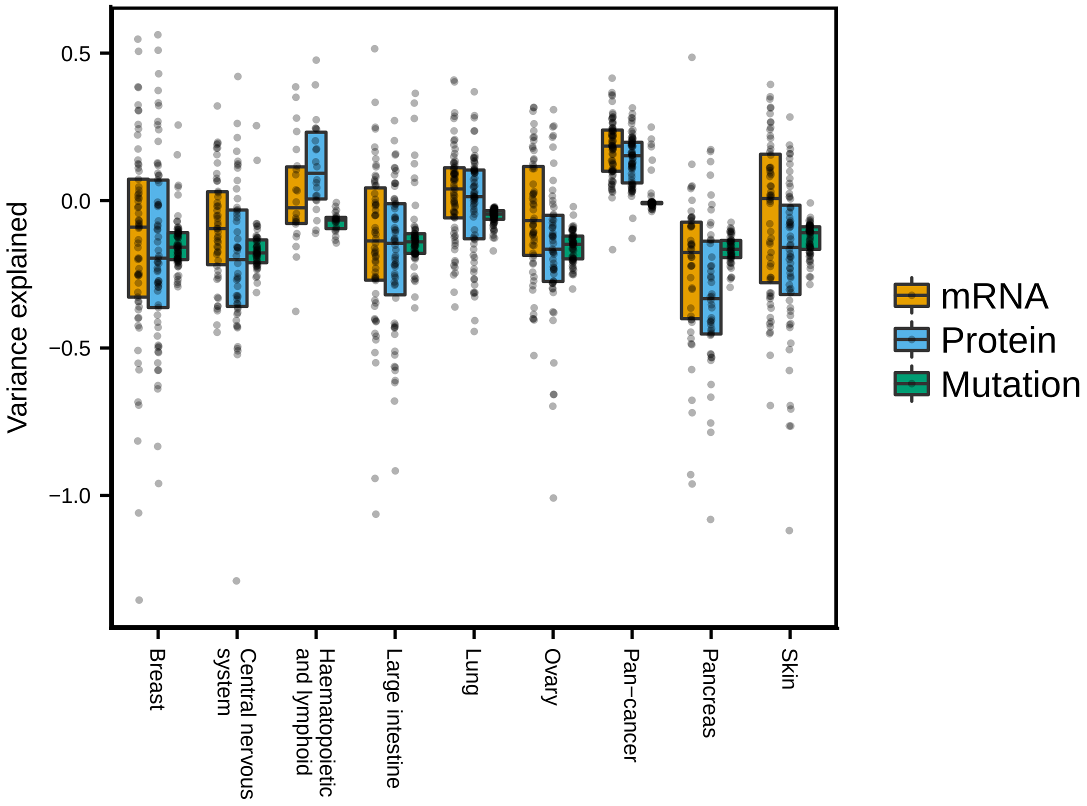

Figure S19: Fraction of variance explained between predicted and measured drug sensitivity after cross-validation using CCLE data (Barretina *et al*, 2012).

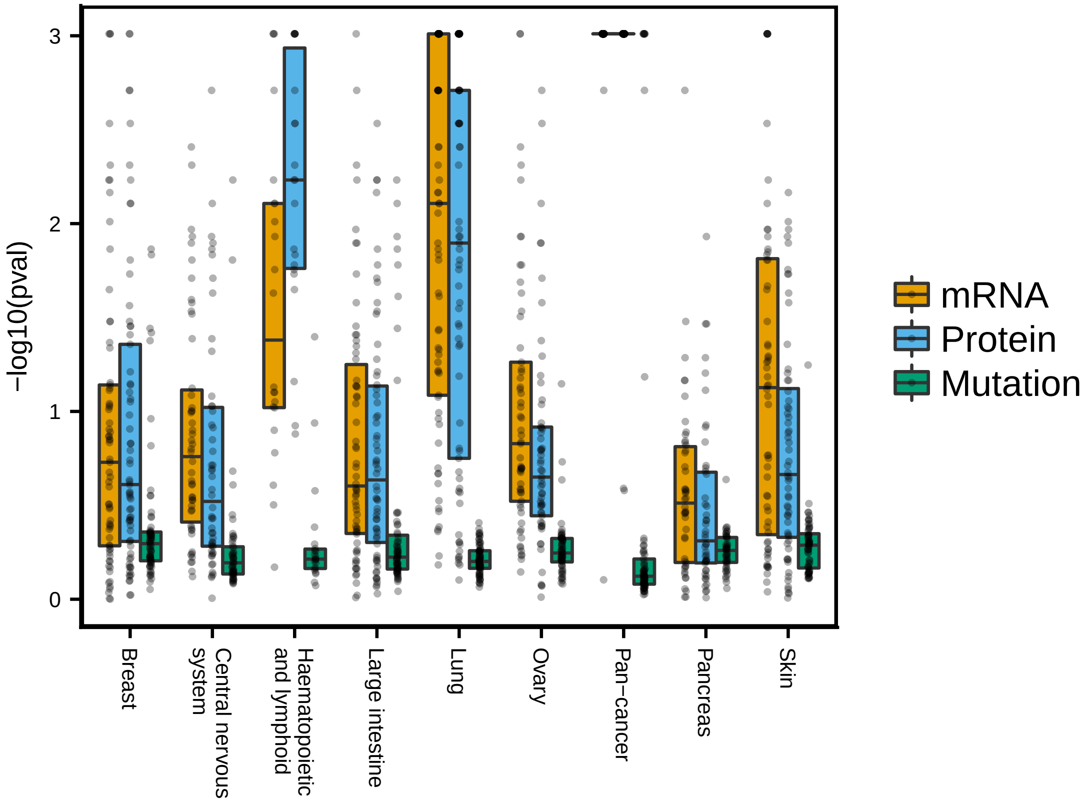

Figure S20: P-values for fraction of variance explained between predicted and measured drug sensitivity after cross-validation using CCLE data (Barretina *et al*, 2012).

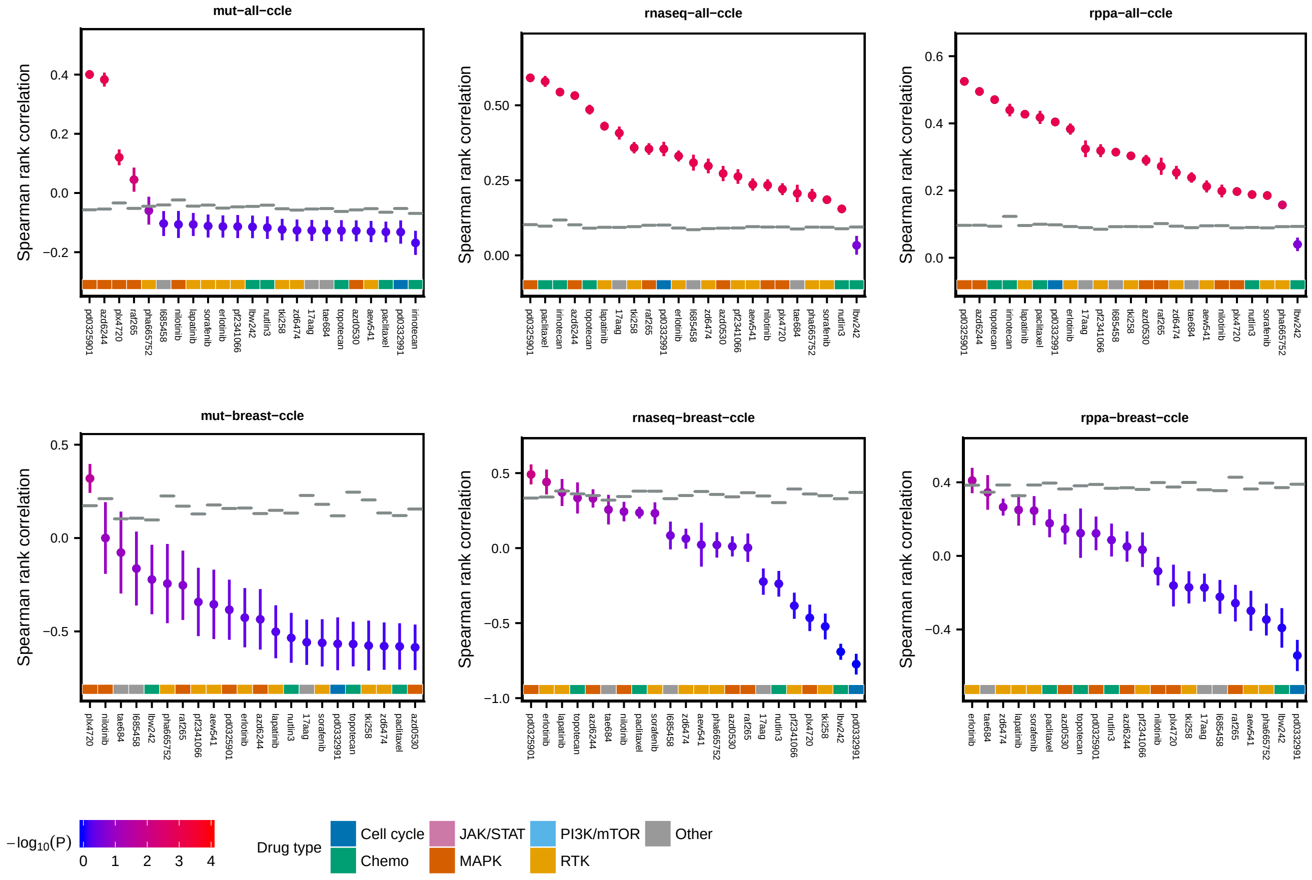

Figure S21: Drug sensitivity predictions using CCLE data (Barretina *et al*, 2012). First part of the figure title show input data: genomic (mut), transcriptomic (rnaseq), proteomic (rppa). Second part of the title shows tissue (all=pan-cancer). Third part of the title shows drug sensitivity data: CTRP (Seashore-Ludlow *et al*, 2015) or CCLE (Barretina *et al*, 2012). Gray bars indicate $P = 0.05$ level of randomized background distributions.

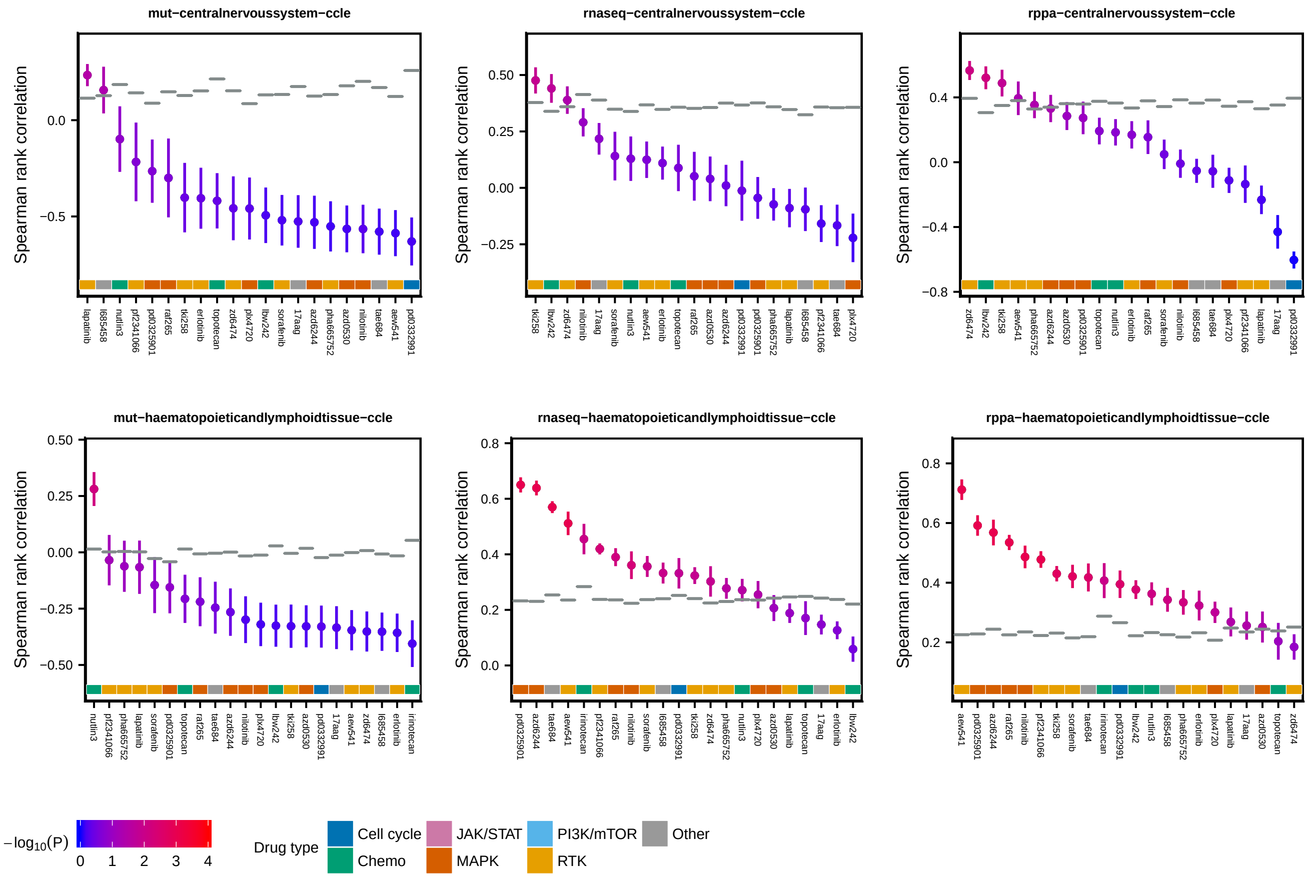

Figure S21: (Cont'd) Drug sensitivity predictions using CCLE data (Barretina *et al*, 2012). First part of the figure title show input data: genomic (mut), transcriptomic (rnaseq), proteomic (rppa). Second part of the title shows tissue (all=pan-cancer). Third part of the title shows drug sensitivity data: CTRP (Seashore-Ludlow *et al*, 2015) or CCLE (Barretina *et al*, 2012). Gray bars indicate $P = 0.05$ level of randomized background distributions.

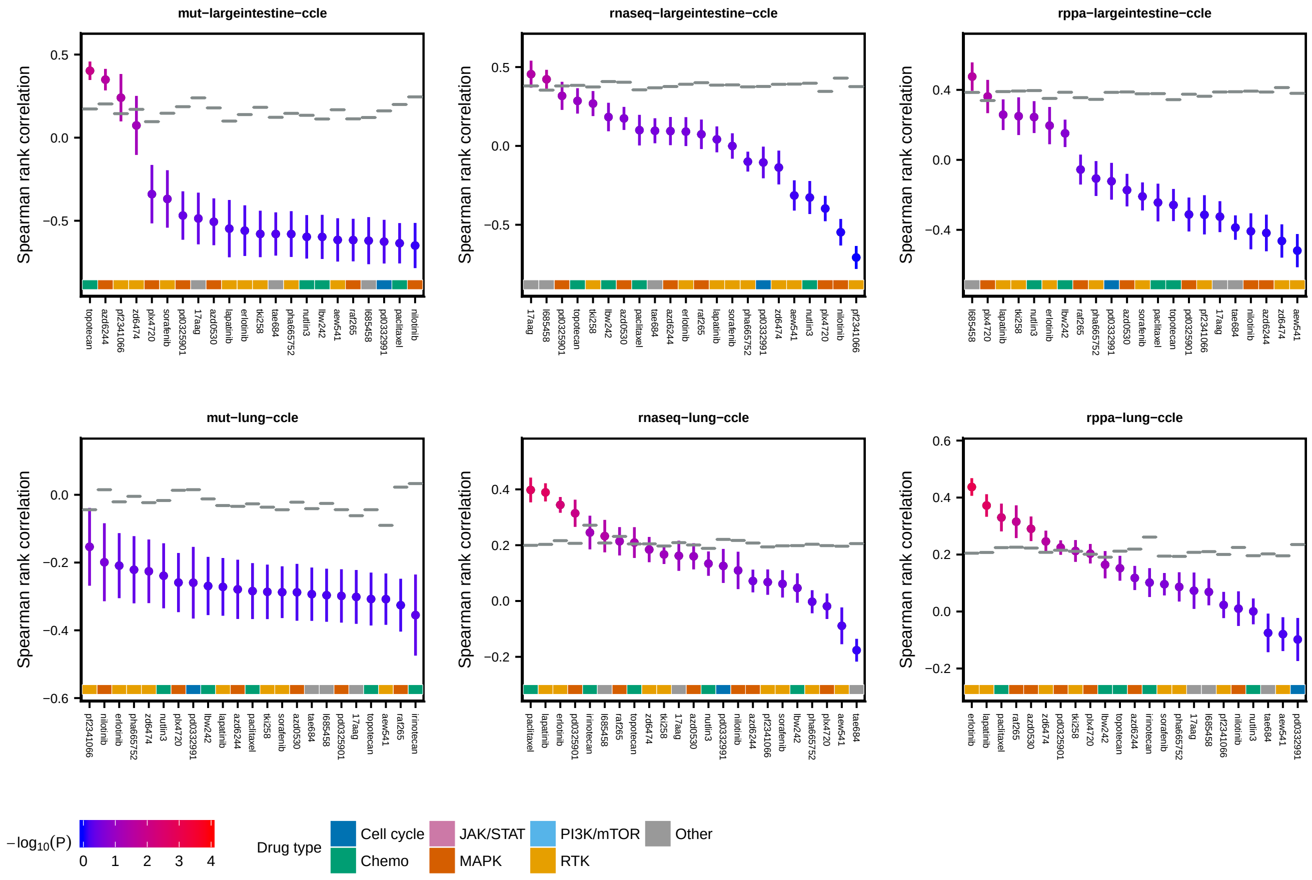

Figure S21: (Cont'd) Drug sensitivity predictions using CCLE data (Barretina *et al*, 2012). First part of the figure title show input data: genomic (mut), transcriptomic (rnaseq), proteomic (rppa). Second part of the title shows tissue (all=pan-cancer). Third part of the title shows drug sensitivity data: CTRP (Seashore-Ludlow *et al*, 2015) or CCLE (Barretina *et al*, 2012). Gray bars indicate $P = 0.05$ level of randomized background distributions.

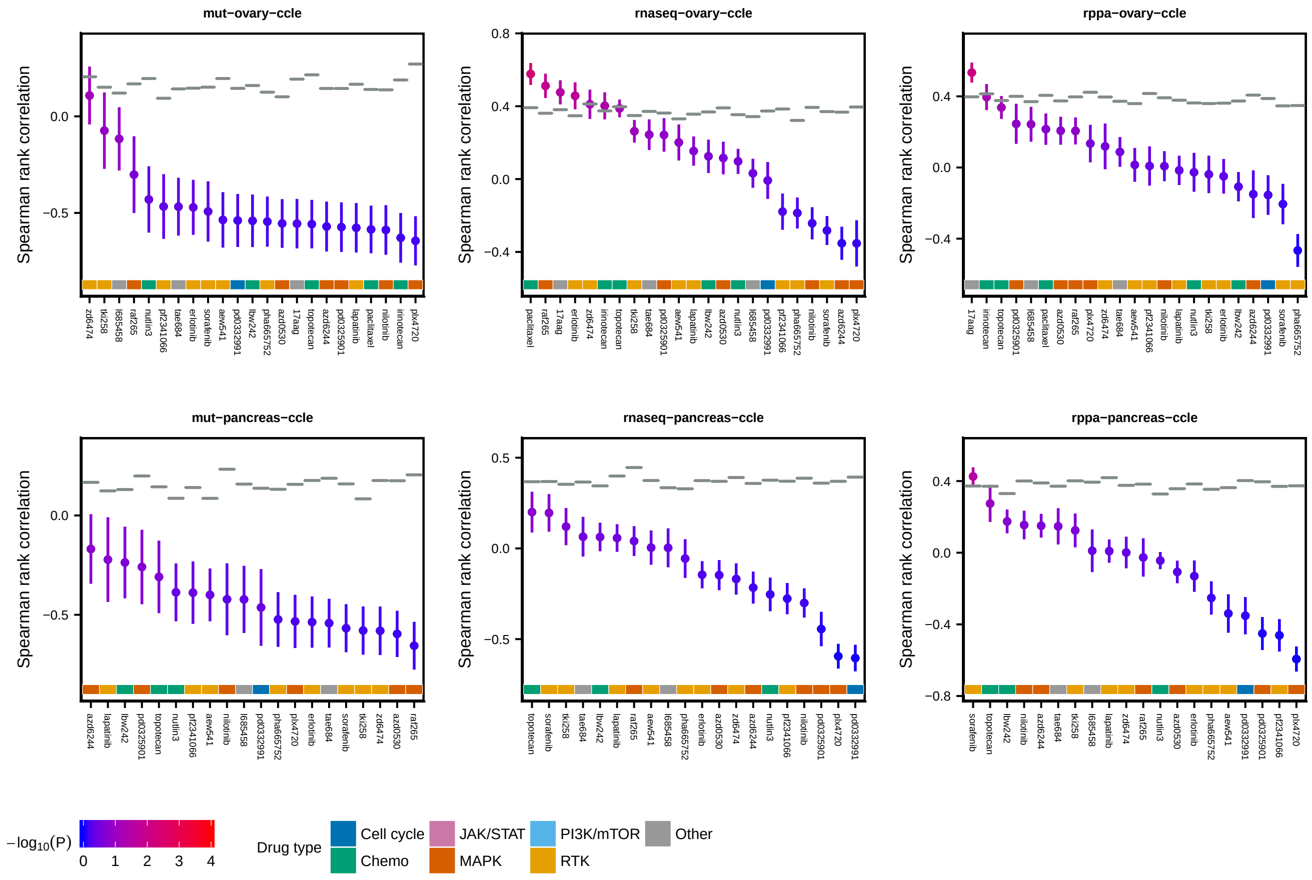

Figure S21: (Cont'd) Drug sensitivity predictions using CCLE data (Barretina *et al*, 2012). First part of the figure title show input data: genomic (mut), transcriptomic (rnaseq), proteomic (rppa). Second part of the title shows tissue (all=pan-cancer). Third part of the title shows drug sensitivity data: CTRP (Seashore-Ludlow *et al*, 2015) or CCLE (Barretina *et al*, 2012). Gray bars indicate $P = 0.05$ level of randomized background distributions.

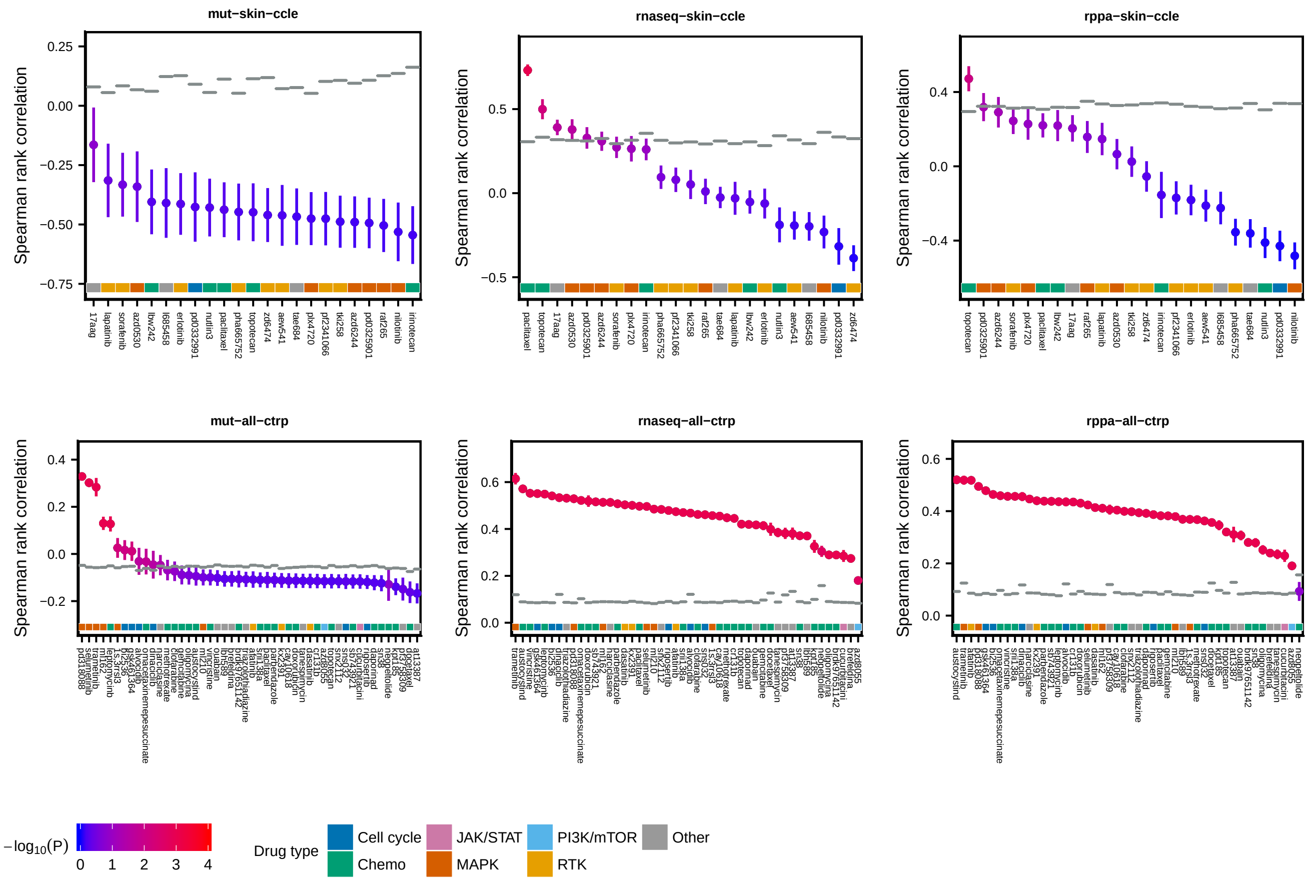

Figure S21: (Cont'd) Drug sensitivity predictions using CCLE data (Barretina *et al*, 2012). First part of the figure title show input data: genomic (mut), transcriptomic (rnaseq), proteomic (rppa). Second part of the title shows tissue (all=pan-cancer). Third part of the title shows drug sensitivity data: CTRP (Seashore-Ludlow *et al*, 2015) or CCLE (Barretina *et al*, 2012). Gray bars indicate $P = 0.05$ level of randomized background distributions.

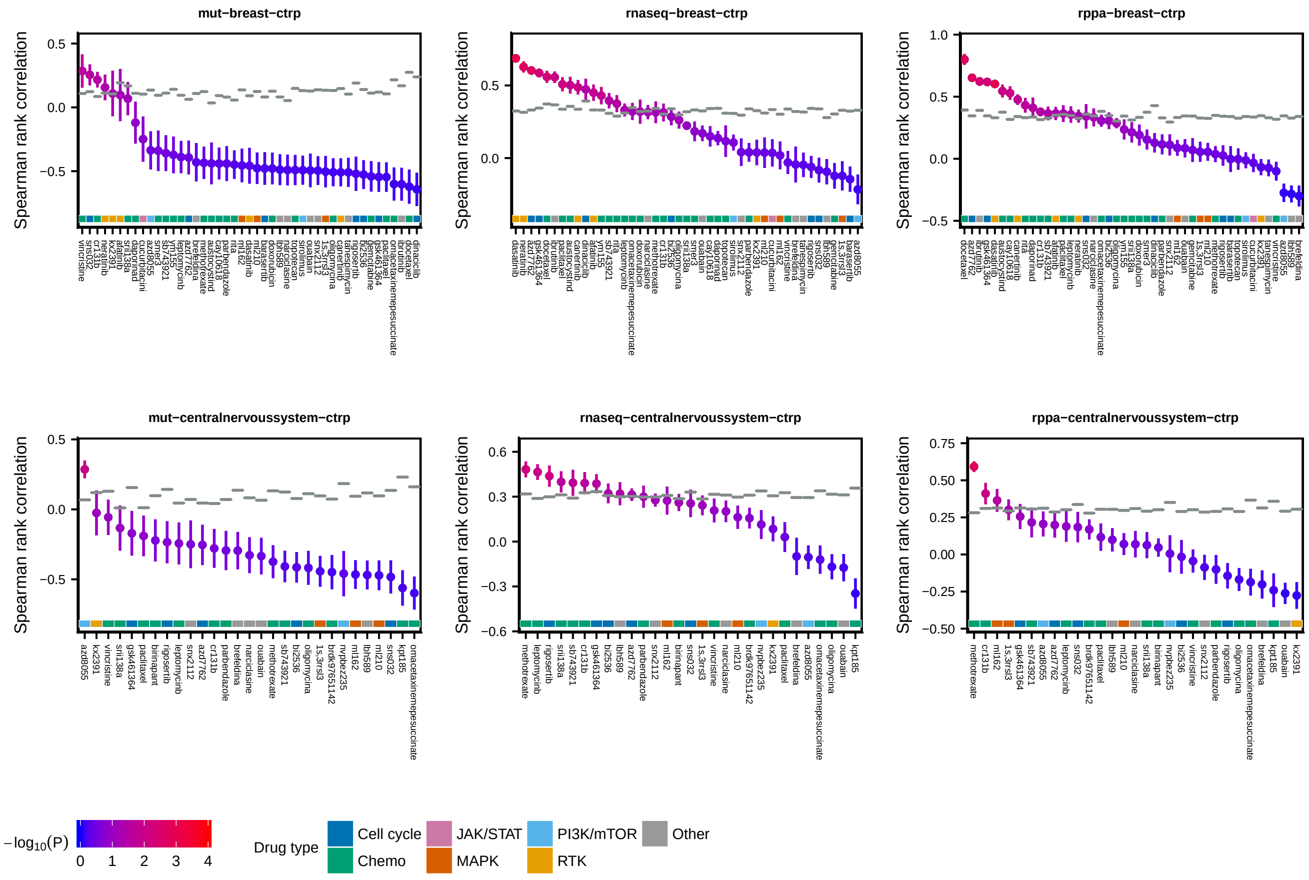

Figure S21: (Cont'd) Drug sensitivity predictions using CCLE data (Barretina *et al*, 2012). First part of the figure title show input data: genomic (mut), transcriptomic (rnaseq), proteomic (rppa). Second part of the title shows tissue (all=pan-cancer). Third part of the title shows drug sensitivity data: CTRP (Seashore-Ludlow *et al*, 2015) or CCLE (Barretina *et al*, 2012). Gray bars indicate $P = 0.05$ level of randomized background distributions.

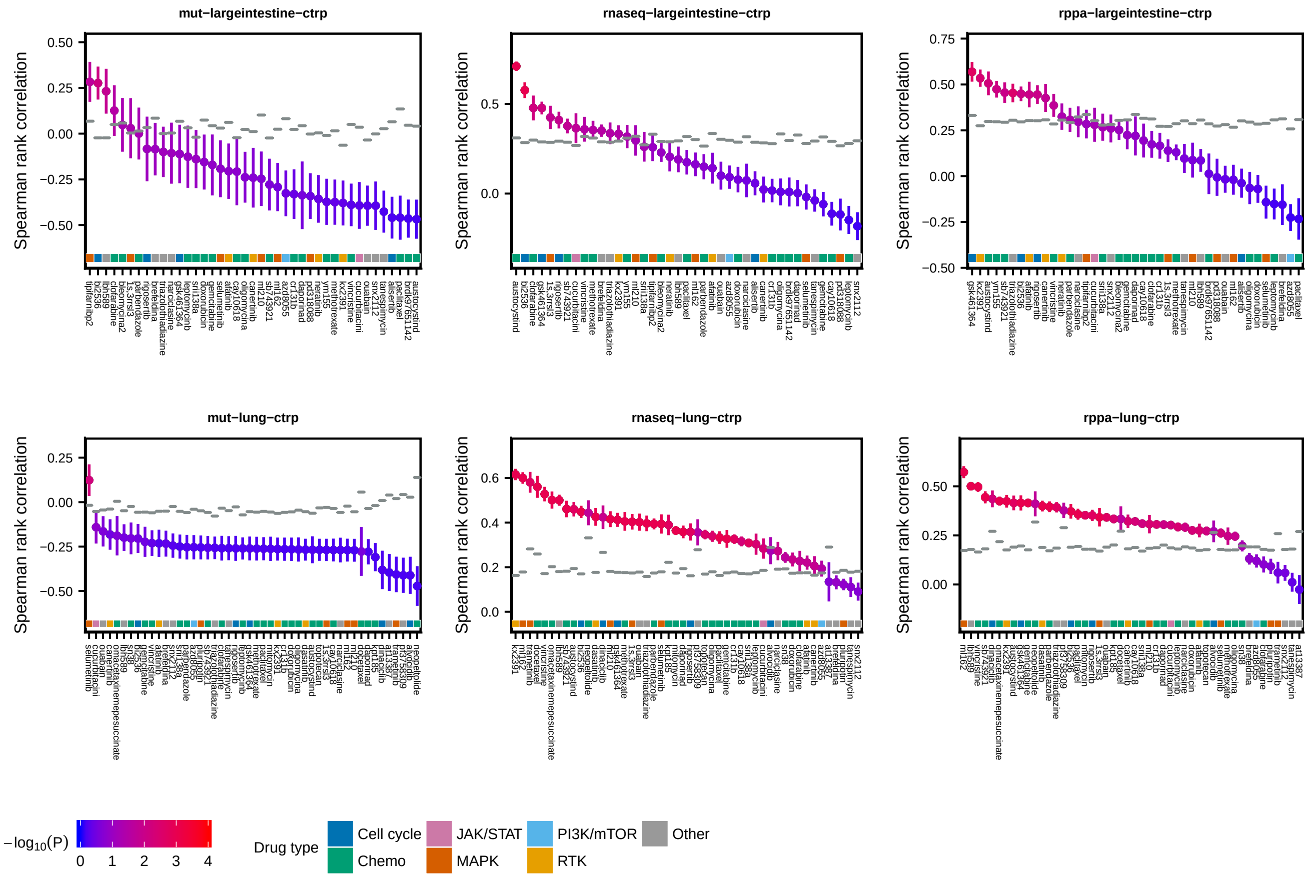

Figure S21: (Cont'd) Drug sensitivity predictions using CCLE data (Barretina *et al*, 2012). First part of the figure title show input data: genomic (mut), transcriptomic (rnaseq), proteomic (rppa). Second part of the title shows tissue (all=pan-cancer). Third part of the title shows drug sensitivity data: CTRP (Seashore-Ludlow *et al*, 2015) or CCLE (Barretina *et al*, 2012). Gray bars indicate $P = 0.05$ level of randomized background distributions.

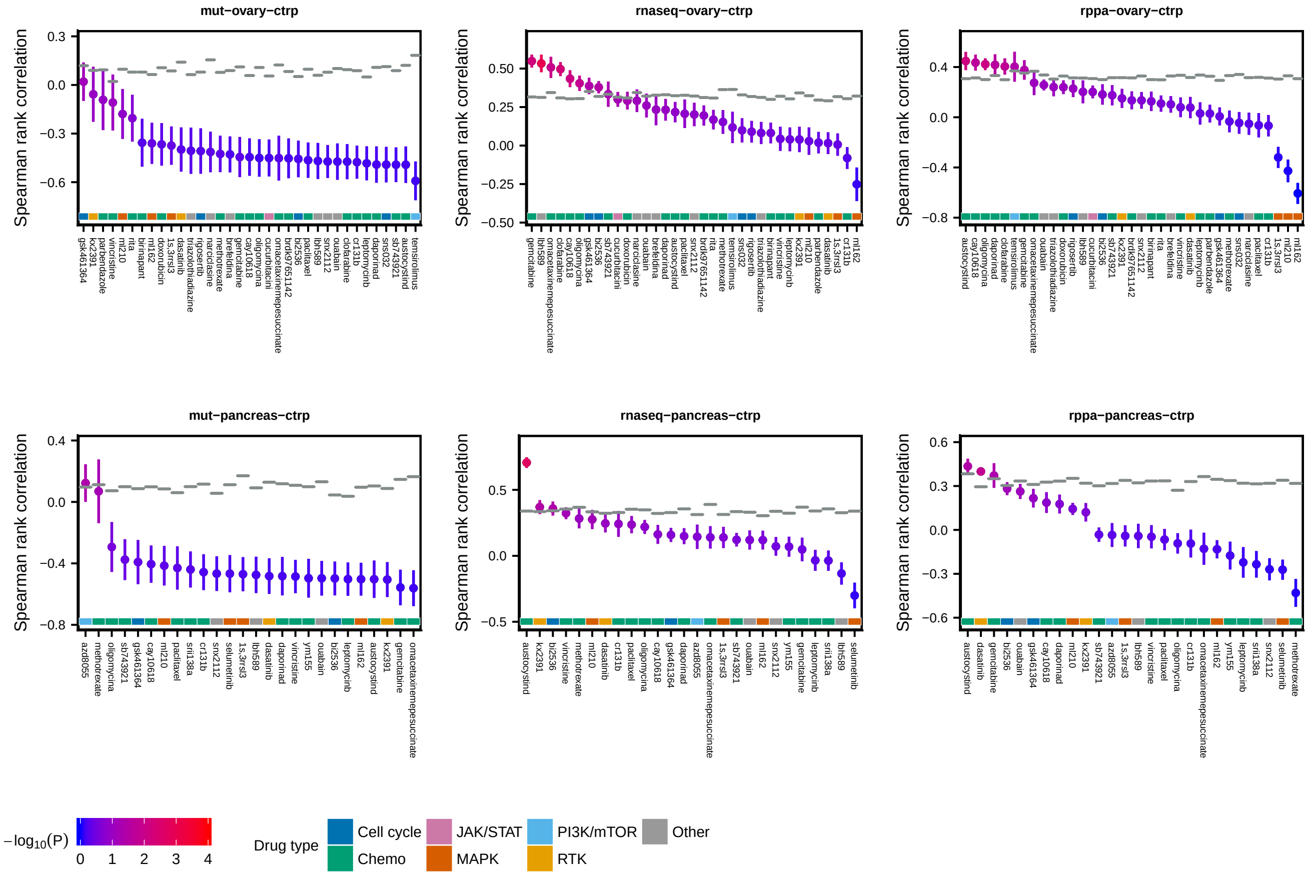

Figure S21: (Cont'd) Drug sensitivity predictions using CCLE data (Barretina *et al*, 2012). First part of the figure title show input data: genomic (mut), transcriptomic (rnaseq), proteomic (rppa). Second part of the title shows tissue (all=pan-cancer). Third part of the title shows drug sensitivity data: CTRP (Seashore-Ludlow *et al*, 2015) or CCLE (Barretina *et al*, 2012). Gray bars indicate $P = 0.05$ level of randomized background distributions.

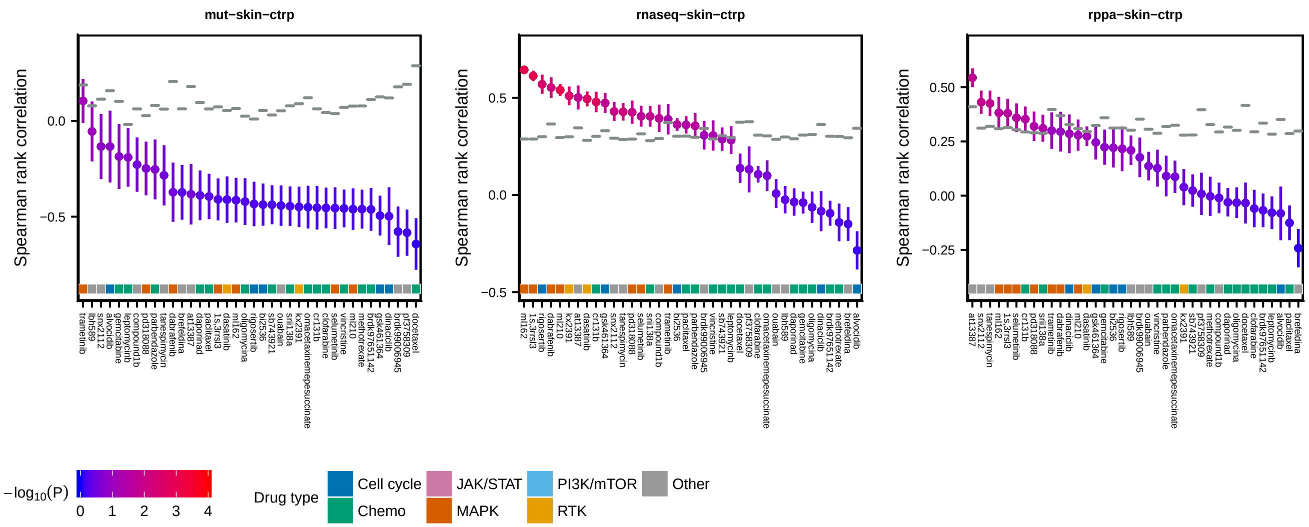

Figure S21: (Cont'd) Drug sensitivity predictions using CCLE data (Barretina *et al*, 2012). First part of the figure title show input data: genomic (mut), transcriptomic (rnaseq), proteomic (rppa). Second part of the title shows tissue (all=pan-cancer). Third part of the title shows drug sensitivity data: CTRP (Seashore-Ludlow *et al*, 2015) or CCLE (Barretina *et al*, 2012). Gray bars indicate $P = 0.05$ level of randomized background distributions.

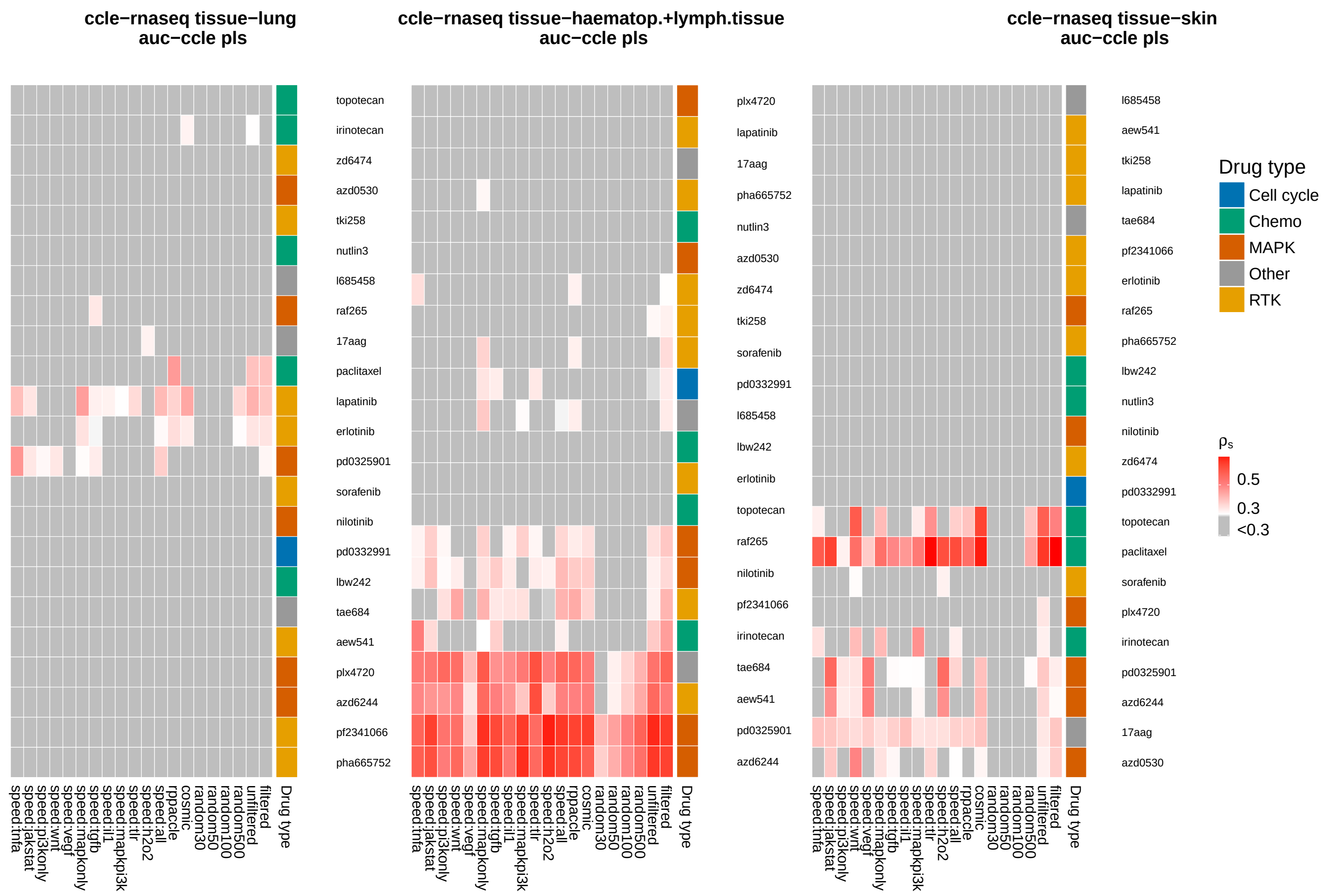

Figure S22: Predicting drug sensitivity from transcriptomic CCLE data (Barretina *et al*, 2012) using genes differentially expressed in key cellular pathways as reported by SPEED (Parikh *et al*, 2010), genes measured in the proteomic dataset, genes in the COSMIC Cancer Gene Census (Forbes *et al*, 2015), randomly selected genes, unfiltered genes, and mean+variance filtered genes.

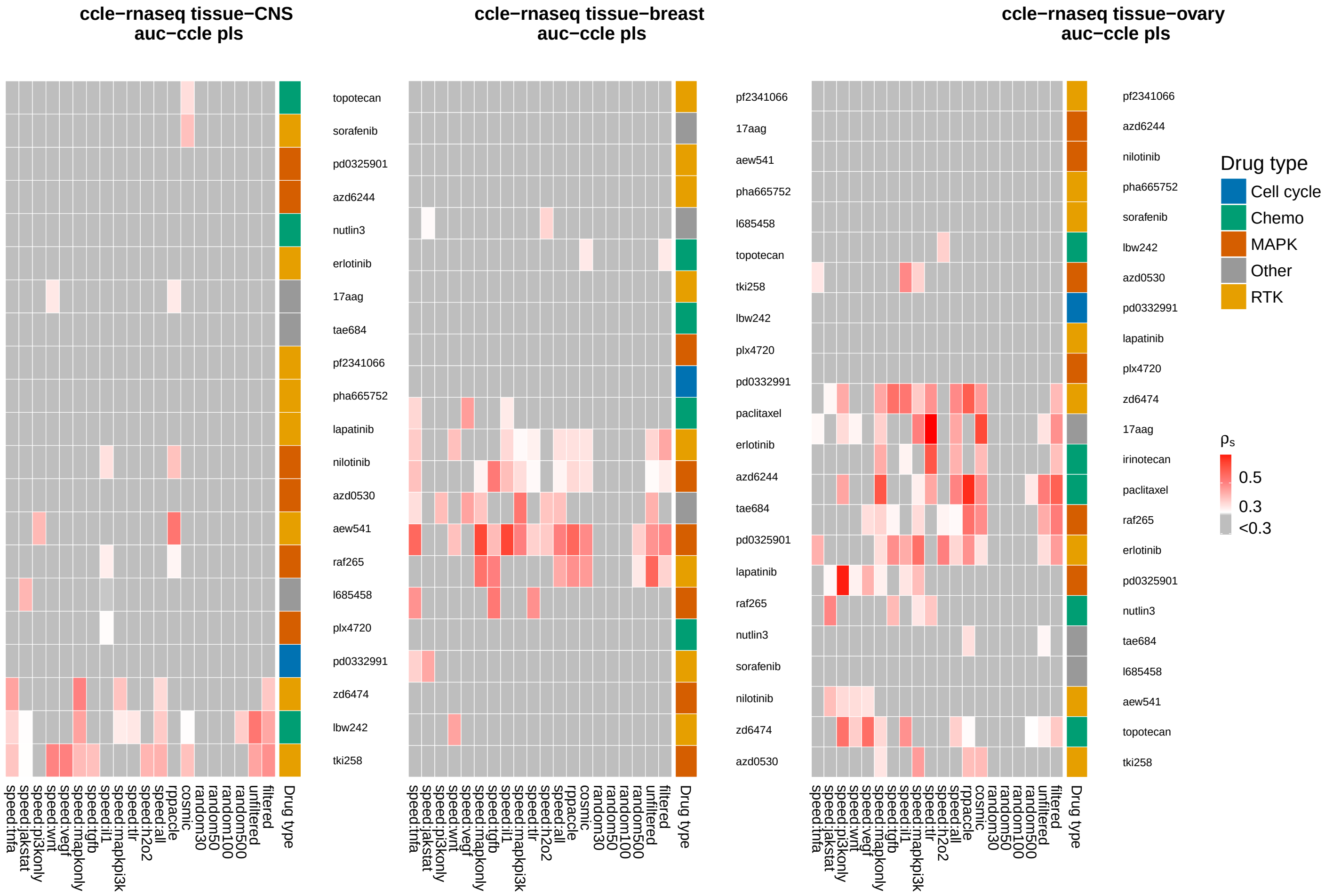

Figure S22: (Cont'd) Predicting drug sensitivity from transcriptomic CCLE data (Barretina *et al*, 2012) using genes differentially expressed in key cellular pathways as reported by SPEED (Parikh *et al*, 2010), genes measured in the proteomic dataset, genes in the COSMIC Cancer Gene Census (Forbes *et al*, 2015), randomly selected genes, unfiltered genes, and mean+variance filtered genes.

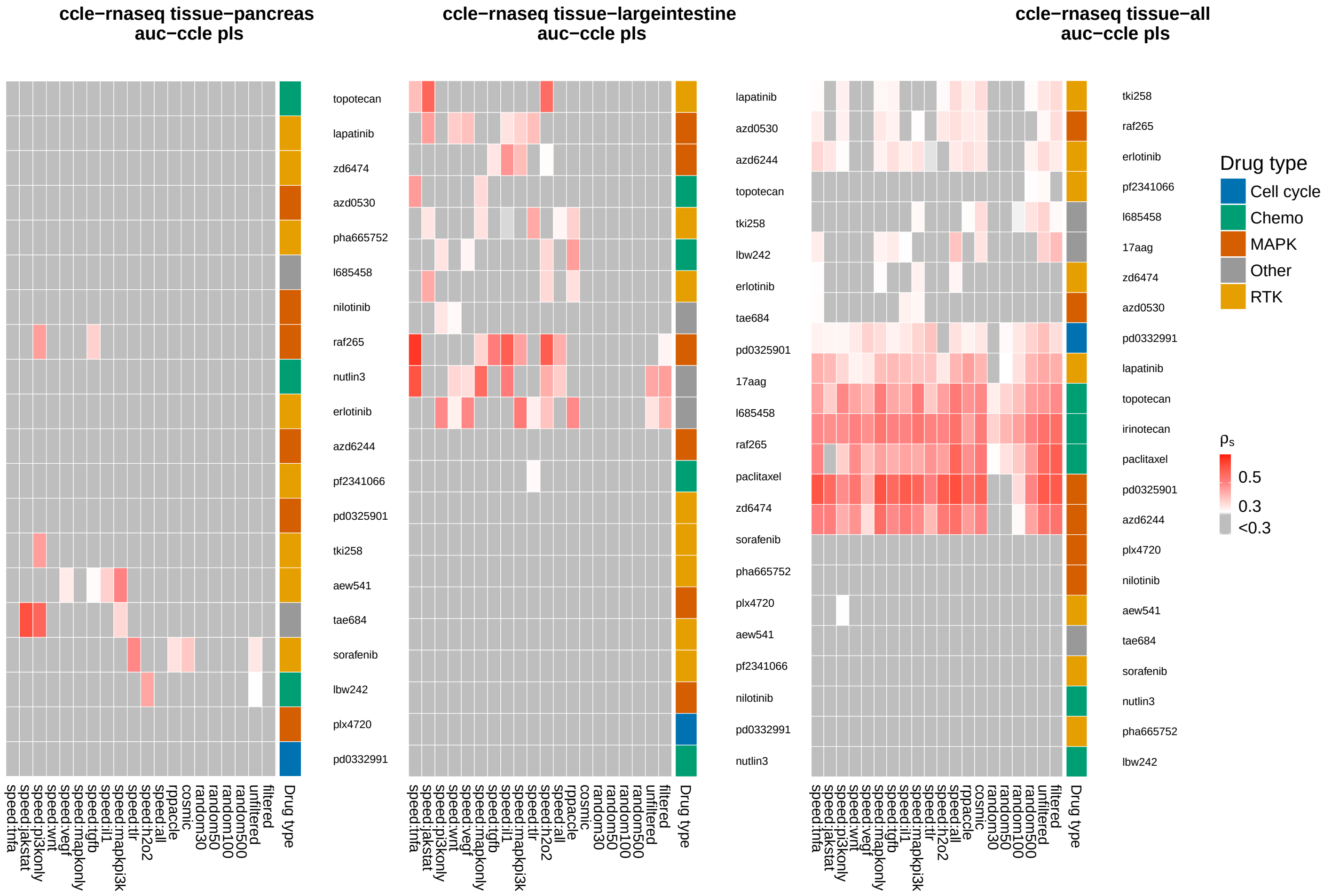

Figure S22: (Cont'd) Predicting drug sensitivity from transcriptomic CCLE data (Barretina *et al*, 2012) using genes differentially expressed in key cellular pathways as reported by SPEED (Parikh *et al*, 2010), genes measured in the proteomic dataset, genes in the COSMIC Cancer Gene Census (Forbes *et al*, 2015), randomly selected genes, unfiltered genes, and mean+variance filtered genes.

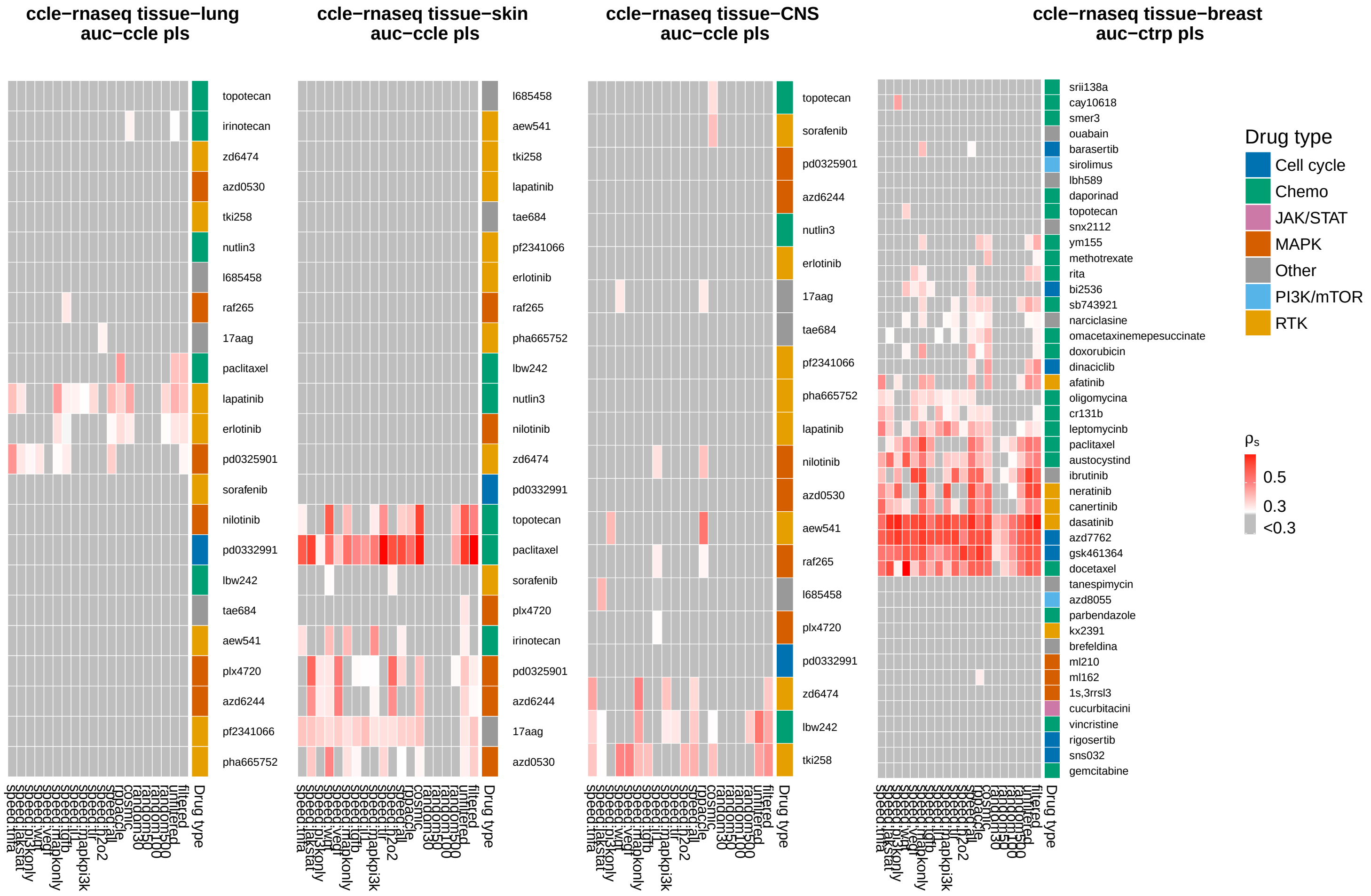

Figure S22: (Cont'd) Predicting drug sensitivity from transcriptomic CCLE data (Barretina *et al*, 2012) using genes differentially expressed in key cellular pathways as reported by SPEED (Parikh *et al*, 2010), genes measured in the proteomic dataset, genes in the COSMIC Cancer Gene Census (Forbes *et al*, 2015), randomly selected genes, unfiltered genes, and mean+variance filtered genes.

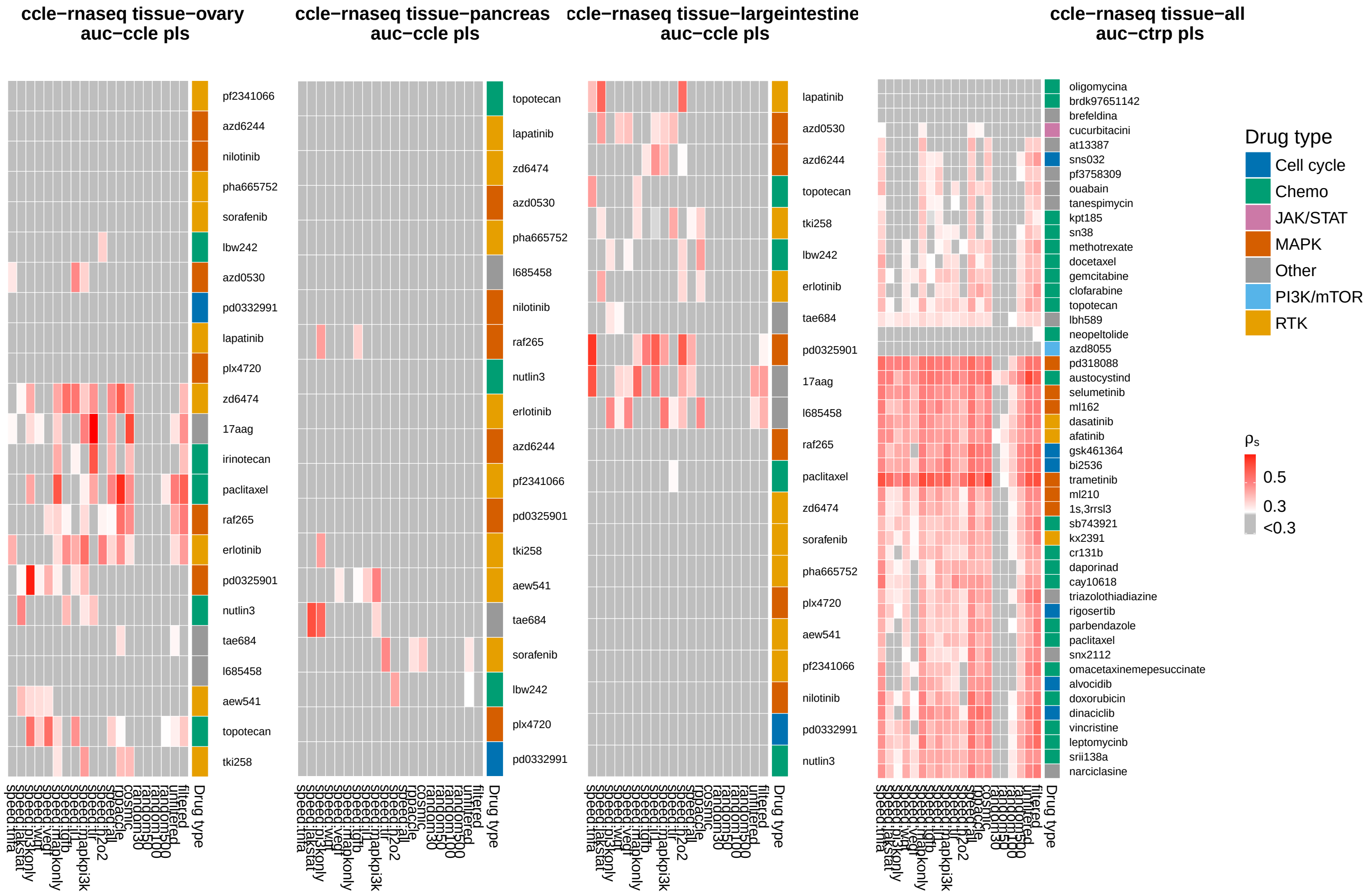

Figure S22: (Cont'd) Predicting drug sensitivity from transcriptomic CCLE data (Barretina *et al*, 2012) using genes differentially expressed in key cellular pathways as reported by SPEED (Parikh *et al*, 2010), genes measured in the proteomic dataset, genes in the COSMIC Cancer Gene Census (Forbes *et al*, 2015), randomly selected genes, unfiltered genes, and mean+variance filtered genes.

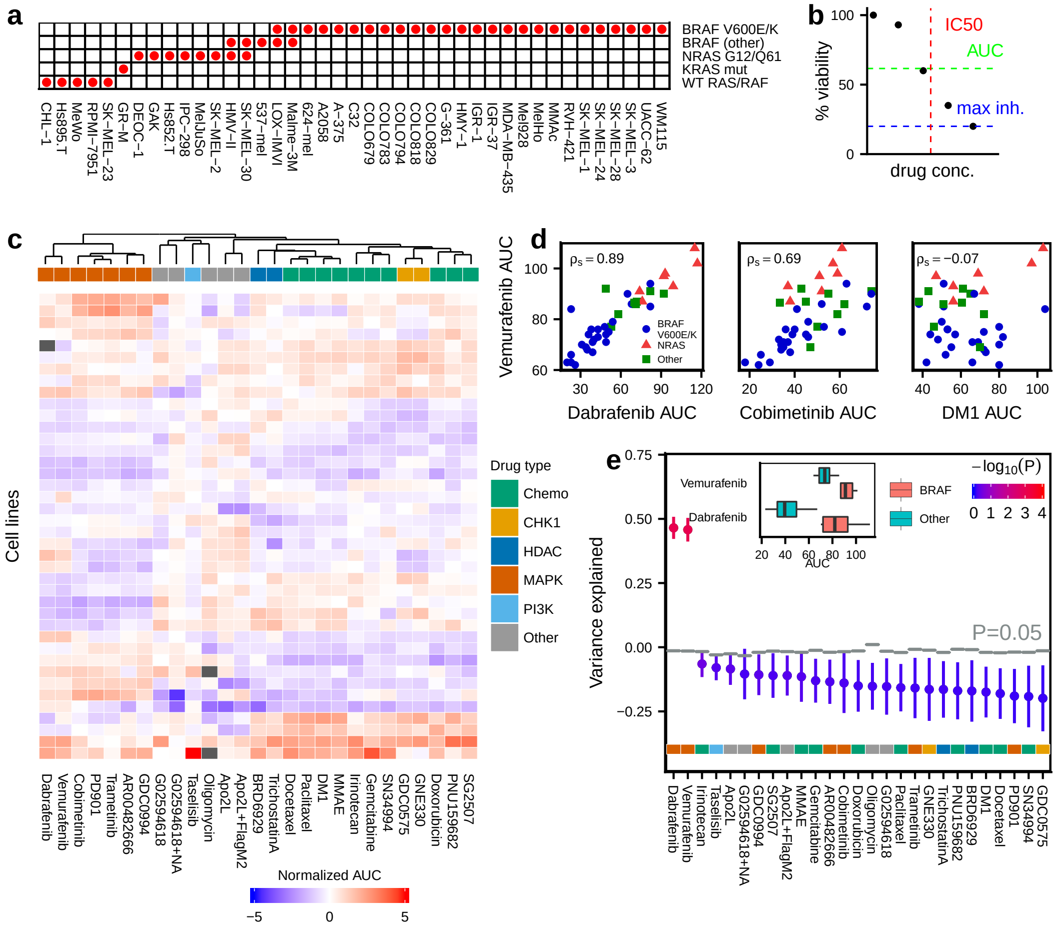

Figure S23: Main figure 1-4 with cell line "duplicates" originating from the same patient removed.

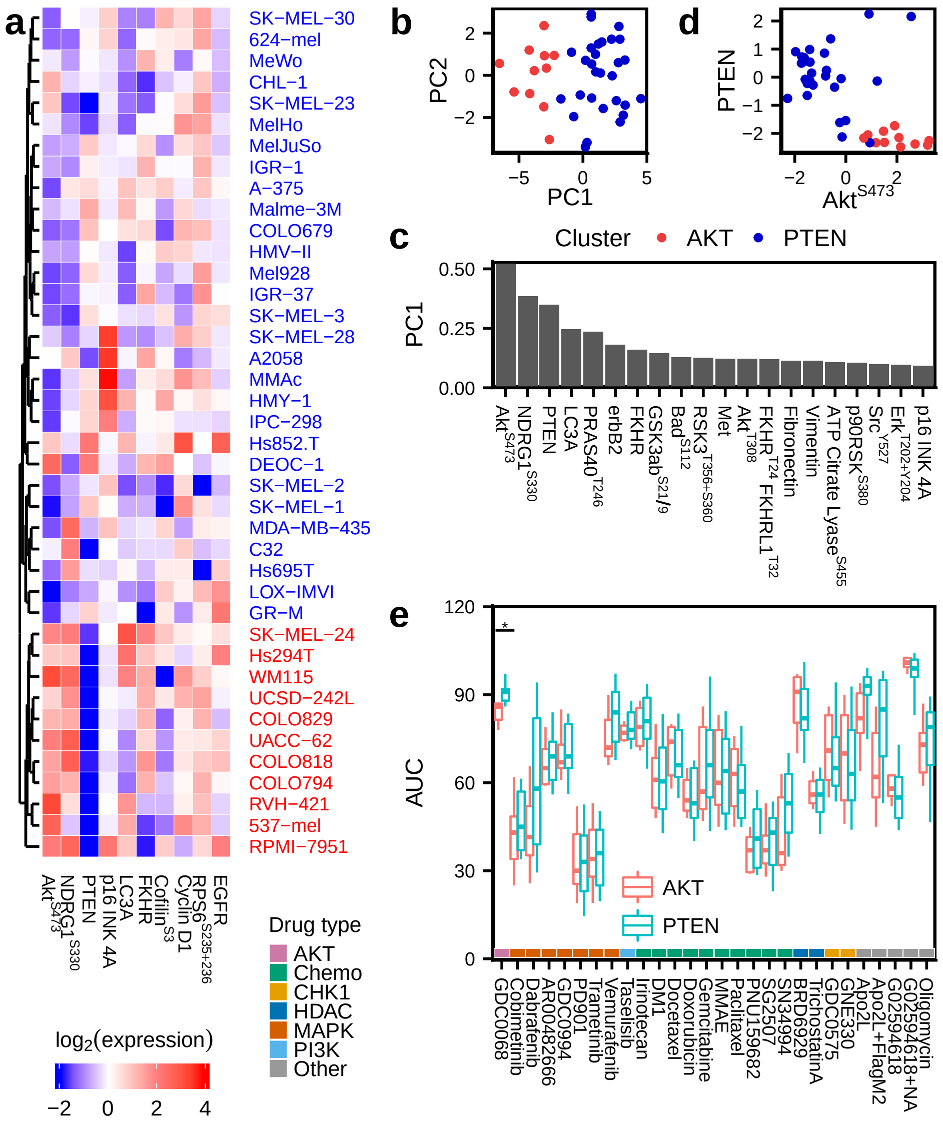

Figure S23: (Cont'd) Main figure 1-4 with cell line "duplicates" originating from the same patient removed.

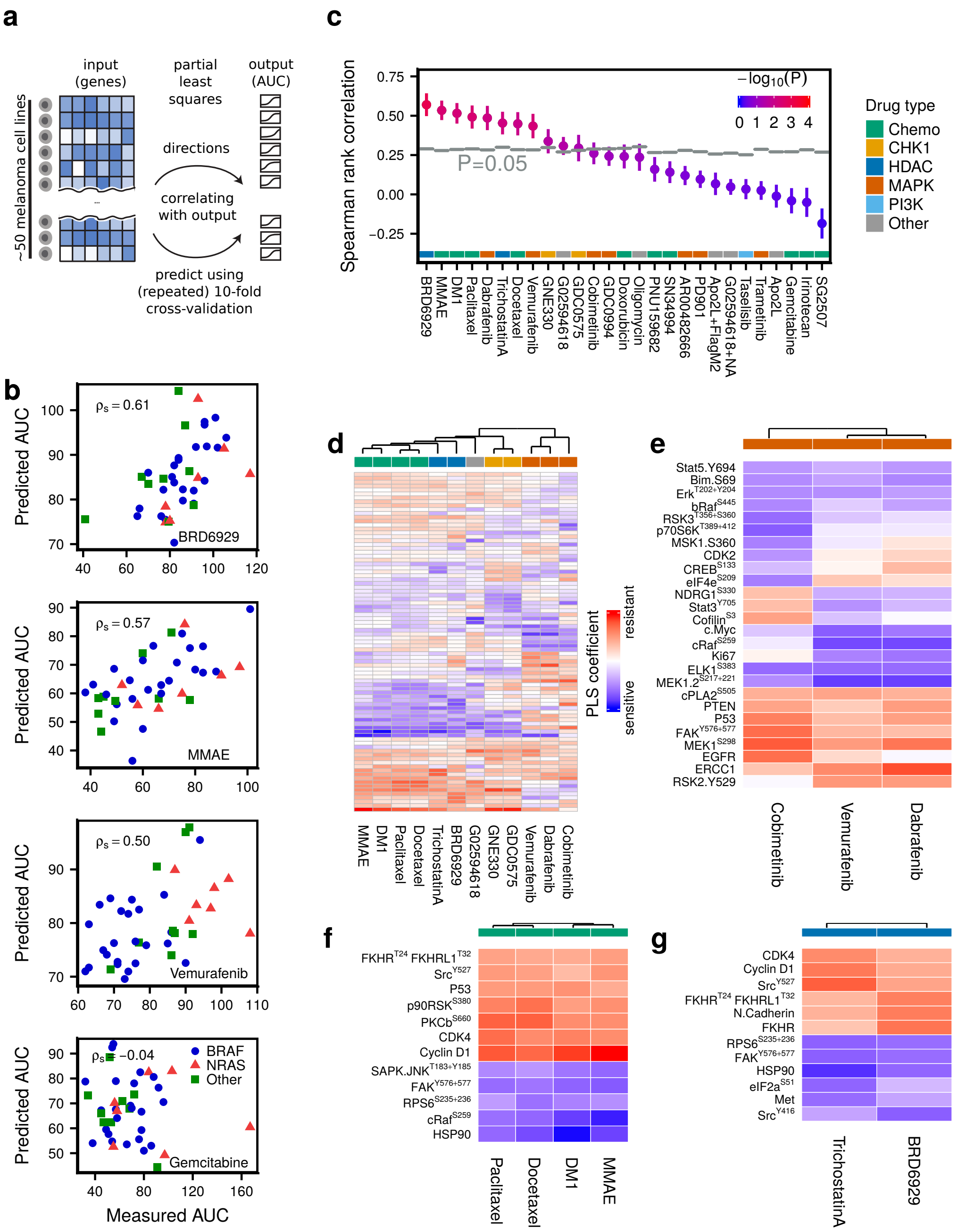

Figure S23: (Cont'd) Main figure 1-4 with cell line "duplicates" originating from the same patient removed.

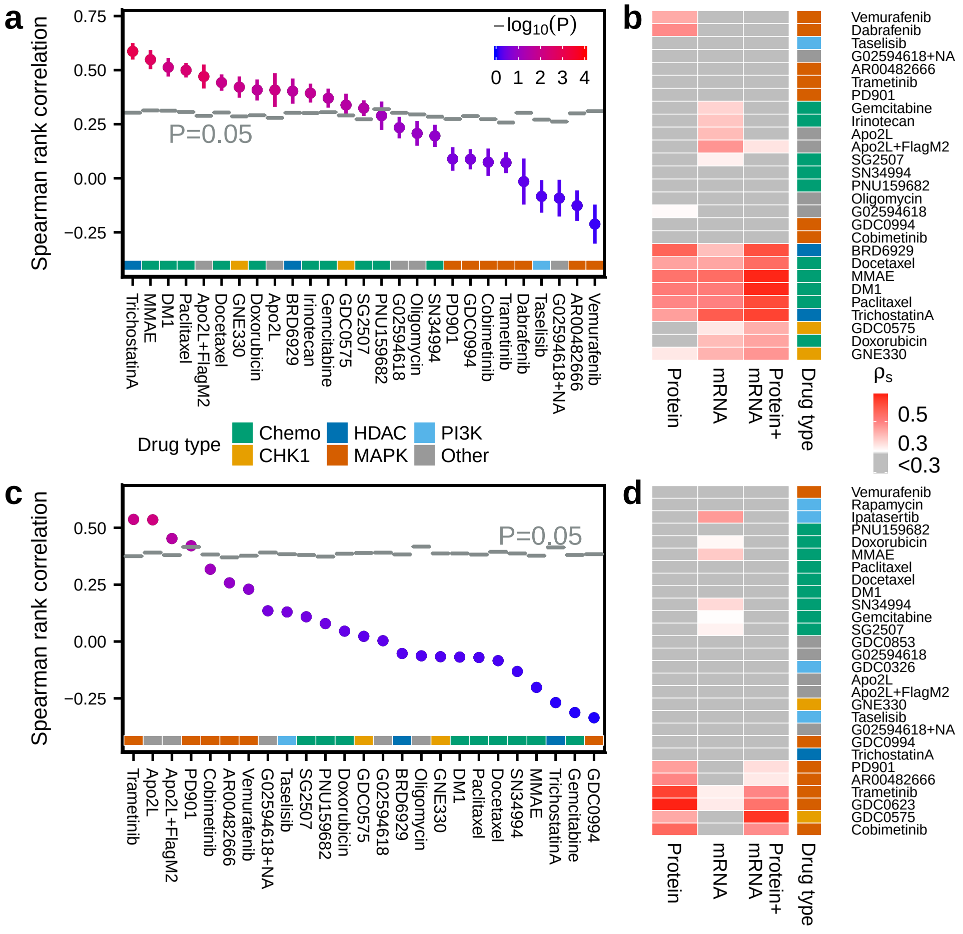

Figure S23: (Cont'd) Main figure 1-4 with cell line "duplicates" originating from the same patient removed.

References

Barretina J, Caponigro G, Stransky N, Venkatesan K, Margolin AA, Kim S, Wilson CJ, Lehár J, Kryukov GV, Sonkin D, Reddy A, Liu M, Murray L, Berger MF, Monahan JE, Morais P, Meltzer J, Korejwa A, Jané-Valbuena J, Mapa FA, *et al* (2012) The Cancer Cell Line Encyclopedia enables predictive modelling of anticancer drug sensitivity. *Nature* **483**: 307–603

Forbes SA, Beare D, Gunasekaran P, Leung K, Bindal N, Boutselakis H, Ding M, Bamford S, Cole C, Ward S, Kok CY, Jia M, De T, Teague JW, Stratton MR, McDermott U, Campbell PJ (2015) COSMIC: exploring the world's knowledge of somatic mutations in human cancer. *Nucleic acids research* **43**: D805—-11

Parikh JR, Klinger B, Xia Y, Marto JA, Bluthgen N (2010) Discovering causal signaling pathways through gene-expression patterns. *Nucleic Acids Research* **38**: W109—-W117

Seashore-Ludlow B, Rees MG, Cheah JH, Coko M, Price EV, Coletti ME, Jones V, Bodycombe NE, Soule CK, Gould J, Alexander B, Li A, Montgomery P, Wawer MJ, Kuru N, Kotz JD, Suk-Yee Hon C, Munoz B, Liefeld T, Dančik V, *et al* (2015) Harnessing connectivity in a large-scale small-molecule sensitivity dataset. *Cancer Discovery* **5**: 1210–1223
