# Supplementary material for: The cancer cell proteome and transcriptome predicts sensitivity to targeted and cytotoxic drugs
Source: Life Sci Alliance. 2019 Jun 28;2(4):e201900445. doi: 10.26508/lsa.201900445 (PMC6600015; doi:10.26508/lsa.201900445)
Supplement: Supplementary file 1 [file Supplementary_Figures_Tables_Data_Sets_and_Source_Codes.zip › LSA-2019-00445_SourceData_01.zip!/Supplement/SourceCode/figures/3_pls_cv.pdf]

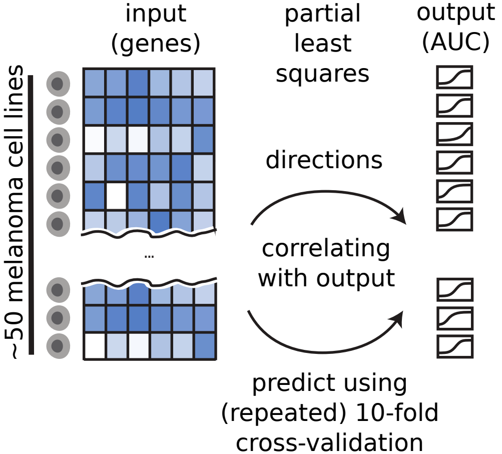
input (genes)
partial least squares
output (AUC)
~50 melanoma cell lines
directions
...
correlating with output
predict using (repeated) 10-fold cross-validation
